# Extracellular matrix-related genes-based prognostic signature for cervical cancer: association of LAMA4 expression with prognosis and response to immunotherapy

**DOI:** 10.3389/fonc.2025.1562115

**Published:** 2025-08-13

**Authors:** Fengchun Gao, Hongyu Shi, Huanhuan Wang, Jin Wang, Dongdong Hao, Yichen Fang

**Affiliations:** ^1^ Department of Gynaecology and Obstetrics, Jinan Maternal and Child Health Care Hospital Affiliated with Shandong First Medical University, Jinan, China; ^2^ College of Health and Elderly Care Industry, Shandong Institute of Commerce and Technology, Jinan, China; ^3^ Prenatal Diagnosis Center, Jinan Maternal and Child Health Care Hospital Affiliated with Shandong First Medical University, Jinan, China; ^4^ Department of Gynecologic Oncology, Jiangsu Cancer Hospital, The Affiliated Cancer Hospital of Nanjing Medical University, Jiangsu Institute of Cancer Research, Nanjing, China

**Keywords:** cervical cancer, extracellular matrix, LAMA4, prognosis, immunotherapy

## Abstract

**Background:**

Extracellular matrix-related genes (ERGs) are crucial in the tumorigenesis of various malignancies, including cervical cancer (CC), but their prognostic significance in CC has not been thoroughly investigated.

**Methods:**

We interrogated RNA-seq expression profiles from the public datasets to identify differentially expressed ERGs. Cox regression analysis was utilized to evaluate their prognostic significance. Consensus clustering classified CC patients into distinct subgroups with varying survival outcomes, immune infiltration, and pathway activation. We constructed an ERGs-based prognostic model using Lasso-Cox regression and examined the expression of LAMA4 in CC by RT-qPCR and immunohistochemistry (IHC), along with its correlation with patient outcomes and response to immunotherapy.

**Results:**

ERGs exhibited significant differential expression between CC and normal tissues, with LAMA4 standing out as a hub gene linked to unfavorable prognosis. Consensus clustering sorted CC patients into two major subgroups with notable differences in survival. A prognostic model consisting of key ERGs robustly predicted overall survival. Evidence from our clinical samples, validated by RT-qPCR and immunohistochemistry (IHC), suggests that elevated LAMA4 expression is significantly associated with poor prognosis and response to immunotherapy.

**Conclusions:**

This investigation underscores the prognostic value of ERGs in CC and identifies LAMA4 as a potential marker for prognosis and immunotherapy response.

## Introduction

Cervical cancer (CC) ranks as a leading cause of cancer-related affliction and death among women globally, particularly in regions with limited healthcare resources (1). In 2021, there were 670,000 new cases of cervical cancer globally, with the highest incidence in low SDI regions. The number of deaths reached 296,667, with the highest mortality also concentrated in low SDI regions. The DALYs caused by cervical cancer were as high as 9.91 million. Cervical cancer incidence peaked between the ages of 40 and 59, with mortality peaking at 55–59 years and DALYs peaking at 50–54 years. The main risk factors for cervical cancer death were unsafe sex and tobacco use (2). While preventative measures such as screening and HPV vaccination have made strides, outcomes for those with advanced CC are still dire, highlighting the critical need for new prognostic indicators and treatment avenues (3, 4). The emergence of immunotherapies targeting PD-L1/PD-1 checkpoints has marked a significant breakthrough in CC management. Yet, the modest response rates to these therapies spotlight the existence of a sizeable group of patients for whom these options are ineffective (5, 6).

At the heart of cellular architecture and function, the extracellular matrix (ECM) not only provides structural integrity but also orchestrates key cellular processes such as communication, differentiation, and migration (7–9). Investigations into the role of ECM-related genes (ERGs) have shed light on their contributions to tumorigenesis, influencing the tumor milieu, blood vessel formation, and the spread of cancer cells (10). Despite these insights, a full understanding of ERGs’ roles in CC, particularly as predictive tools or therapeutic targets, is still evolving.

Our research endeavored to fill the existing voids by establishing an extensive atlas of ERGs in CC, utilizing RNA-seq data from the TCGA-CESC collection and GEO compilations. We crafted a prognostic framework predicated on ERGs, with a particular emphasis on scrutinizing the role of LAMA4. Complementing this, the examination of clinical samples from CC patients at the Jinan Maternal and Child Health Care Hospital, a branch of Shandong First Medical University (SFMU), further corroborated the close correlation between LAMA4 expression and both prognostic outcomes and the efficacy of immunotherapeutic interventions.

## Materials and methods

### Data collection and analysis of extracellular matrix-related genes in CC

We retrieved RNA-seq data and pertinent clinical information for CC patients from the TCGA-CESC project and supplemented these with GEO datasets (e.g., GSE63514, GSE14404). A distinctive feature of the TCGA database is that it contains long-term follow-up data on patients. Detailed clinical information of the TCGA-CESC cohort is shown in **Supplementary Table 1**. A comprehensive list of ECM-related genes (ERGs) was curated from the literature. We identified significant differential expression using stringent criteria: an FDR below 0.05 and a log2FC exceeding 1. Visualization of these changes was achieved through heatmaps and volcano plots, while GO and KEGG analyses elucidated the functional roles and pathways of the ERGs.

### Assessing ERGs’ prognostic value in CC

We appraised the influence of ERGs on CC patient survival, drawing on TCGA-CESC and GEO data. Using R’s “survival” package, univariate Cox regression pinpointed ERGs with notable prognostic implications. We depicted the expression interplay of these ERGs with a network diagram.

### ERGs expression clustering and survival division

We contrasted ERG expression in CC against controls, classifying CC patients into clusters by their expression signatures through “ConsensusClusterPlus.” Kaplan-Meier plots analyzed survival variances among clusters. PCA, UMAP, and tSNE validated our clustering, while “GSVA” and “GSEABase” explored KEGG pathway discrepancies across clusters. We also examined the links between gene expression patterns and clinical attributes in CC patients.

### Establishing an ERGs-centric prognostic model

We identified prognostically relevant ERGs via univariate Cox regression in the “survival” package. These ERGs were integrated into a Lasso-Cox model using “glmnet,” creating a prognostic index defined by ∑yβyfinedti where i is the ERG count. Lasso regression honed the model’s predictive precision. A heatmap showcased the association between risk scores and ERG signatures.

Patients were sorted into high- or low-risk categories based on median prognostic index scores, with Kaplan-Meier analysis contrasting their survival. ROC curves’ AUC values appraised the model’s predictive capacity.

### Determination and confirmation of central ERGs

We investigated differentially expressed ERGs in the STRING database to construct a PPI network. Cytoscape and cytoHubba’s MCC algorithm ranked ERGs, identifying key candidates through a Venn diagram. The highest MCC scorer was selected as the central ERG. We then connected the central ERG’s expression to overall survival (OS) and progression-free survival (PFS) across subgroups.

### Exploring immune dynamics and pathways linked to central ERG

We probed the TIMER database for immune cell infiltration data, analyzing correlations with genes and Tracking Tumor Immunophenotype (TIP) scores using spearman analysis, presented via the linkET package. CancerSEA’s database was employed to gauge pathway activities, with Pearson correlations mapping the central ERG’s relationships with gene set scores.

### Single-cell analysis of LAMA4

We analyzed single-cell RNA-seq data (GSE168652) to gauge LAMA4 expression levels. “Seurat” performed sample selection and quality checks, while “SingleR” handled clustering and subtype identification. UMAP distilled the data into 2D heatmaps, and ggplot2 visualized gene expression across cell types.

### Association of LAMA4 with immunotherapy efficacy

We performed an evaluative study on the expression levels of LAMA4 in the context of a broad immunotherapy expression profile. The analysis categorized patients into response groups (R), including Complete Response (CR) and Partial Response (PR), and Non-Response groups (NR) such as Stable Disease (SD) and Progressive Disease (PD), plotted against the z-score transformed expression levels of LAMA4.

The IMvigor210 study, a critical phase II clinical trial, assessed the therapeutic effectiveness and safety profile of atezolizumab, an antagonist of the PD-L1 immune checkpoint, in subjects with advanced or metastatic urothelial carcinoma (11). Atezolizumab’s mechanism involves inhibiting the PD-L1 and PD-1 receptor interaction on T cells, potentially empowering the immune response against tumor cells. We rigorously investigated LAMA4 expression under these treatment conditions to deduce its viability as a predictive biomarker for immunotherapeutic efficacy.

### Spatial deconvolution analysis of LAMA4

Employing deconvolution strategies, we determined the cellular composition at each tissue slide location, adhering to strict quality metrics for single-cell transcriptomic data, including gene expression counts, unique molecular identifiers (UMIs), and mitochondrial RNA content. Utilizing CIBERSORTx for enrichment score matrices and Seurat for spatial visualization through SpatialFeaturePlot, we depicted cellular abundance. To explore the relationship between cellular distribution and LAMA4 expression, we applied Spearman correlation analysis, presented via the linkET package.

### LAMA4 expression validation by reverse transcription-quantitative polymerase chain reaction

We validated the detected expression of LAMA4 in our prognostic model across CC and normal tissue samples using RT-qPCR. Following the manufacturer’s protocol, we isolated total RNA and synthesized cDNA from 500 ng of RNA using the PrimeScriptTM RT kit. PCR amplification was performed with primers specific to LAMA4 and GAPDH, and the 2−(ΔΔCt) method was employed to quantify LAMA4 expression levels.

### Immunohistochemistry analysis of LAMA4

Our study included 73 CC patients from SFMU, with surgical procedures taking place between January 2018 and December 2023. Eligibility criteria encompassed histologically confirmed CC, no pre-surgical adjuvant therapy, and patients were over 18 years old, with comprehensive clinical and pathological data. Ethical adherence was confirmed by the hospital’s Ethics Committee (Approval ID: KY R-24-149), with informed consent from all subjects. Detailed patient clinical characteristics are presented in **Table 1** and **Supplementary Table 2**. Moreover, 55 patients underwent ICI therapy (Chemotherapy combined with pembrolizumab), with outcomes evaluated via the iRECIST v1.1 criteria (**Supplementary Table 3**).

**Table 1:** Correlation between clinicopathological features and LAMA4 expression in our hospital cohort.

Clinical features		Total:73(100.0%)	LAMA4 expression		*P*-value*
			Negative:23 (31.51%)	Positive:50 (68.49%)	
Age	<60	15 (20.55%)	5 (6.85%)	10 (13.70%)	
	≥60	58 (79.45%)	18 (24.66%)	40 (54.79%)	1.0000
Gender	Male	0 (0.00%)	0 (0.00%)	0 (0.00%)	
	Female	73 (100.00%)	23 (31.51%)	50 (68.49%)	1.0000
T	T1	27 (36.99%)	16 (21.92%)	11 (15.07%)	
	T2-T4	46 (63.01%)	7 (9.59%)	39 (53.42%)	0.0002*
N	N0	61 (83.56%)	22 (30.14%)	39 (53.42%)	
	N1	12 (16.44%)	1 (1.37%)	11 (15.07%)	0.0887
M	M0	65 (89.04%)	22 (30.14%)	43 (58.90%)	
	M1	8 (10.96%)	1 (1.37%)	7 (9.59%)	0.4218
FIGO	I	27 (36.99%)	16 (21.92%)	11 (15.07%)	
	II-IV	46 (63.01%)	7 (9.59%)	39 (53.42%)	0.0002*

*Fisher’s exact test. Bold values indicate statistical significance (*P* < 0.05).

IHC staining used a specific LAMA4 antibody on paraffin-embedded tissue sections, with two independent pathologists assessing staining intensity and pattern. A score was computed from intensity and extent of staining, with scores ≥c considered positive. These IHC results were then analyzed in relation to clinical outcomes and ICI therapy responses.

### Statistical analysis

We utilized R software (version 4.0.2) for statistical analyses, considering p-values <0.05 as significant. Two-group comparisons were made with the Student’s t-test, while one-way ANOVA was used for multi-group analyses. We adjusted for multiple comparisons to maintain the integrity of our results. Data from TCGA and GEO were harmonized by correcting for batch effects, ensuring result reliability and precision.

## Results

### Delineating ERGs expression patterns in CC

**Figure 1:**
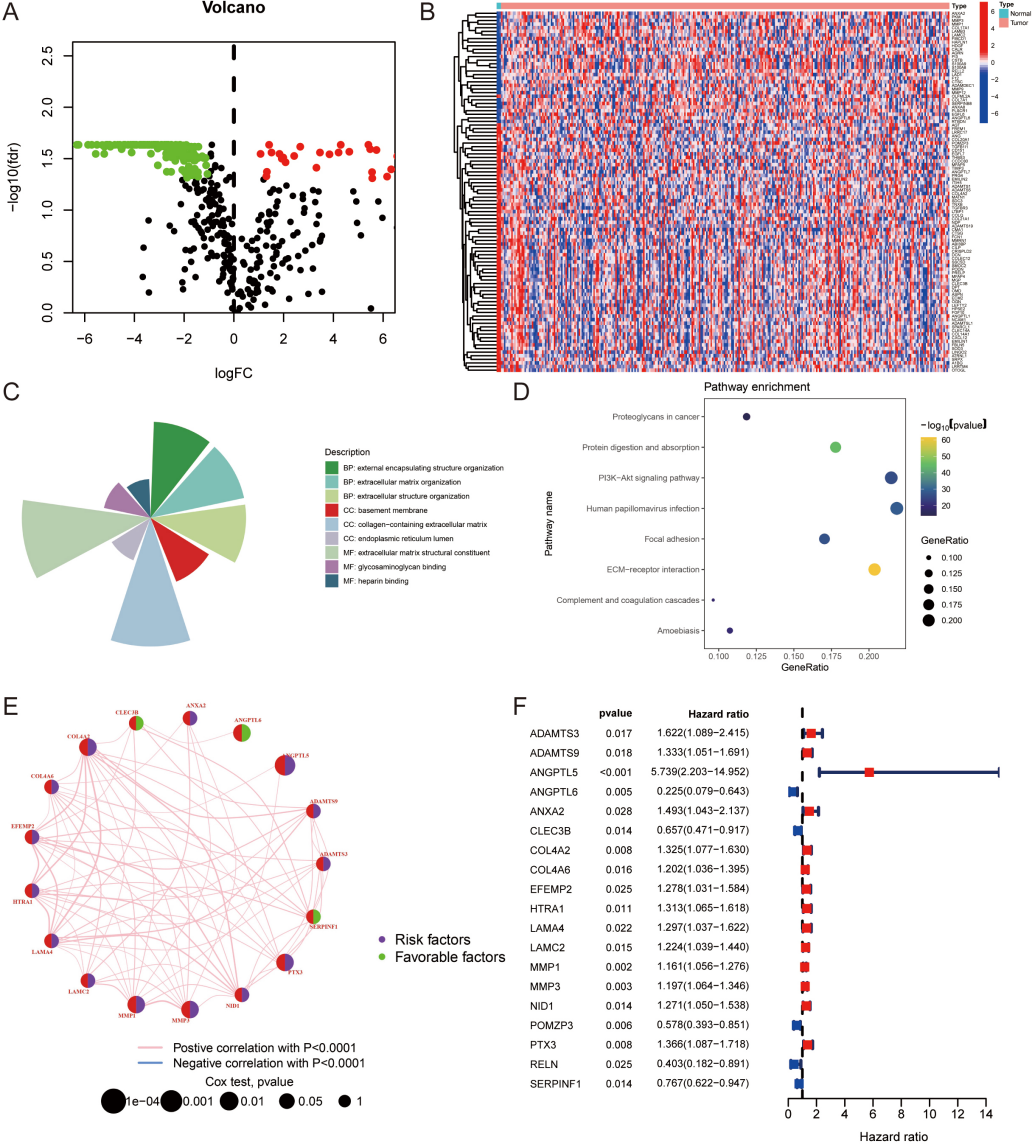
Expression patterns and functional analysis of ERGs in cervical cancer: **(A)** Volcano plot illustrating the differential expression of ERGs in cervical cancer, highlighting genes with statistically significant discrepancies in expression levels. **(B)** Heatmaps depicting the differential expression of ERGs within the TCGA-CESC dataset, showcasing distinct expression profiles. Top upregulated and top downregulated differentially expressed ERGs, sorted by log2FC. **(C)** Gene Ontology (GO) enrichment analysis results, showing a concentration of ERGs in biological processes related to “external encapsulating structure organization,” cellular components such as “collagen-containing extracellular matrix,” and molecular functions including “extracellular matrix structural constituent.” Circle maps complement the GO enrichment findings. **(D)** KEGG pathway analysis revealing significant enrichment of ERGs in the “ECM-receptor interaction” pathway, indicating their involvement in ECM structural and functional complexities. **(E)** The interactions among ERGs are depicted in a network diagram. **(F)** The ERGs that correlate with the prognostic outcomes in CC patients.

We assembled an exhaustive inventory of 568 ERGs based on existing literature (refer to **Supplementary Table 4** for details). The pattern of gene expression discrepancies is depicted in a volcano plot (**Figure 1A**). Following this, differential expression analysis using ERGs within the TCGA-CESC dataset was conducted and illustrated through heatmaps (**Figure 1B**).

Gene Ontology (GO) enrichment analysis was performed on the selected gene sets, uncovering a notable concentration of ERGs in the “external encapsulating structure organization” within the biological process (BP) domain. Analysis of cellular components (CC) revealed a significant presence of ERGs in the “collagen-containing extracellular matrix”, and the chief molecular function (MF) identified for these ERGs was “extracellular matrix structural constituent”, as outlined in **Figure 1C**. Circle maps further delineate the GO enrichment results. Pathway analysis via KEGG revealed a marked enrichment in the “ECM-receptor interaction” pathway, detailed in **Figure 1D**. These analyses indicate that ERGs are integral to the structural and functional complexities of the ECM.

The interactions among ERGs are depicted in a network diagram (**Figure 1E**), and **Figure 1F** displays the ERGs that correlate with the prognostic outcomes in CC patients.

### Categorization of CC subtypes through consensus clustering

**Figure 2:**
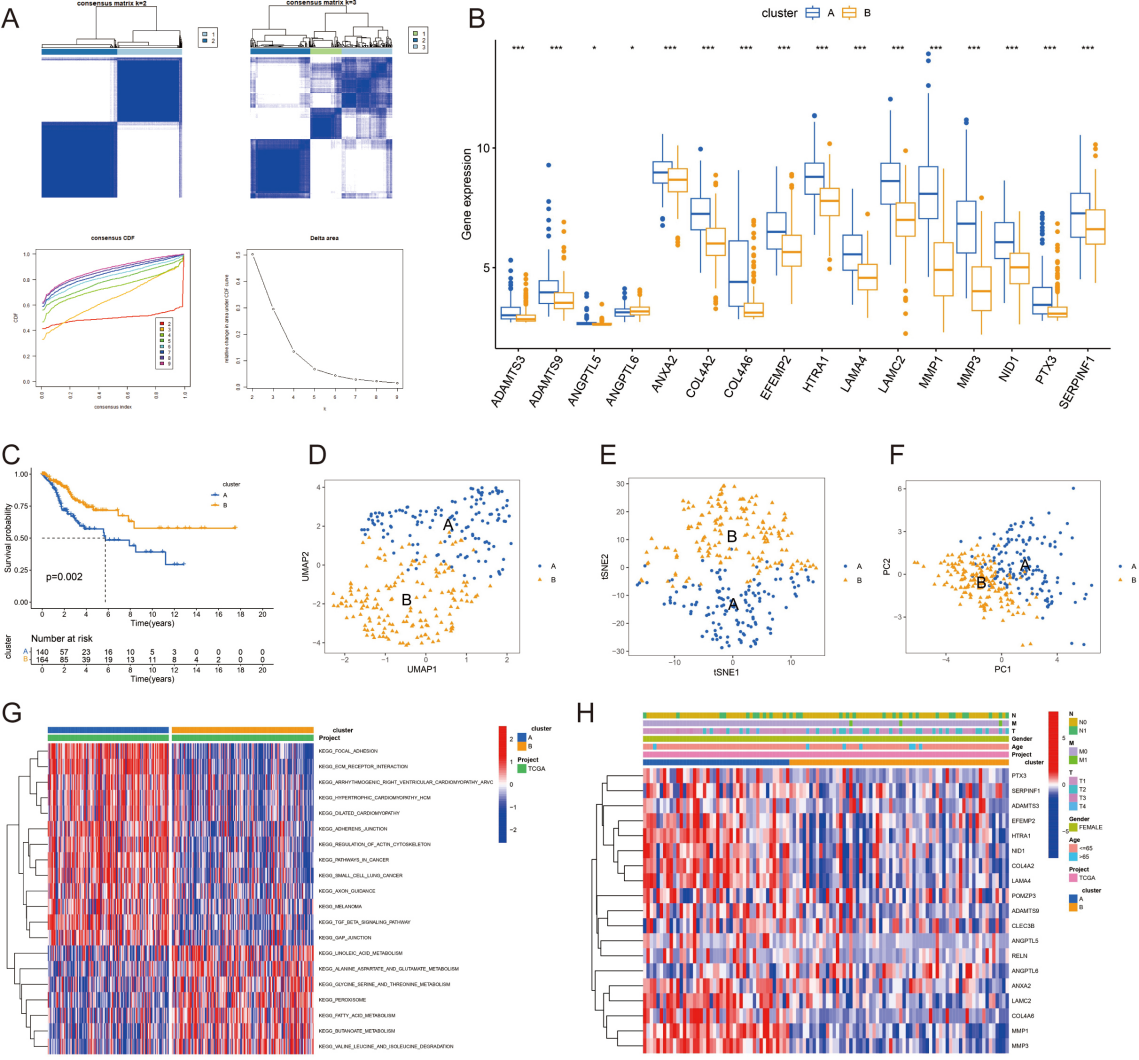
Consensus clustering of cervical cancer subtypes and prognostic analysis: **(A)** Consensus clustering of the TCGA-CESC dataset identifying two distinct molecular subtypes (k=2) of cervical cancer. **(B)** Gene expression profiles of the identified subtypes, highlighting differential expression patterns. **(C)** Kaplan-Meier survival curves comparing the overall survival (OS) between the two subtypes, with cluster A demonstrating poorer survival outcomes. **(D-F)** Validation of the subtype distinction using UMAP, TSNE, and PCA analysis, confirming the separation between subgroups. **(G)** KEGG pathway activations unique to each subtype, with cluster A associated with pathways indicative of poor prognosis. **(H)** Correlation of gene expression patterns with clinical attributes in cervical cancer patients. *P < 0.05; ***P < 0.001.

In an effort to stratify CC patients into molecular subtypes, consensus clustering was employed, followed by Kaplan-Meier survival curves to assess the prognostic variance between the clusters. Using the “ConsensusClusterPlus” R package, we determined the ideal number of clusters to be two (k=2), as depicted in **Figure 2A**. The gene expression profiles across the subgroups are displayed in **Figure 2B**. This division indicated distinct survival probabilities, with cluster A showing poorer survival in the OS analysis (p=0.002, **Figure 2C**). Techniques such as UMAP, TSNE, and PCA confirmed the distinct separation between the two subgroups (**Figures 2D–F**).

We identified specific KEGG pathway activations that differed between clusters A and B (**Figure 2G**), with a full list available in **Supplementary Table 5**. The findings suggest that Cluster A, which is linked to worse prognoses, is characterized by increased activity in pathways such as KEGG_FOCAL_ADHESION, KEGG_ECM_RECEPTOR_INTERACTION, and KEGG_PATHWAYS_IN_CANCER. We also examined the links between gene expression patterns and clinical attributes in CC patients (**Figure 2H**).

### Development and evaluation of an ERGs-based prognostic framework

**Figure 3:**
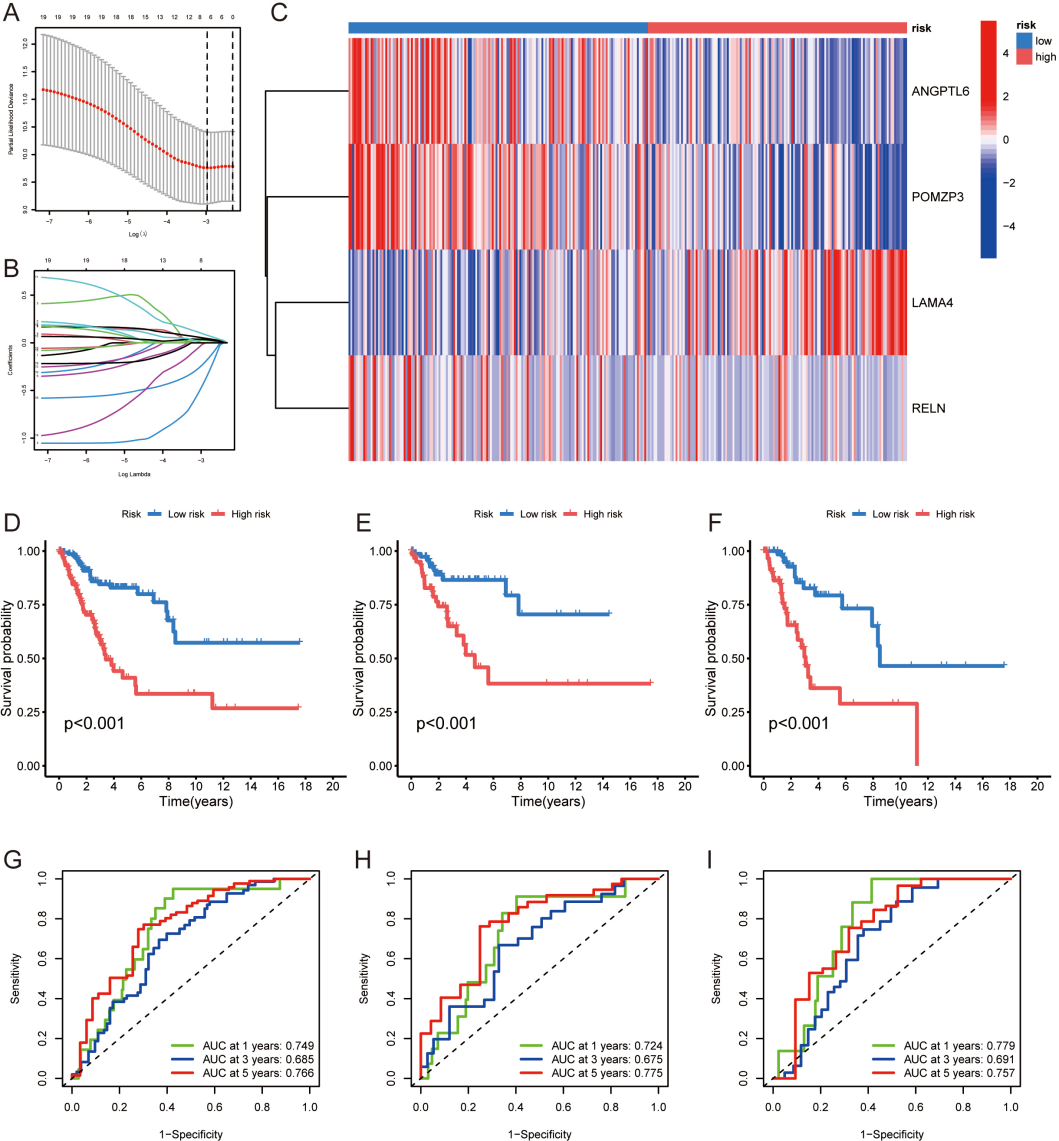
Development of an ERGs-based prognostic model for cervical cancer: **(A, B)** Lasso regression analysis used to derive a prognostic model consisting of 4 ERGs (ANGPTL6, POMZP3, LAMA4, RELN). **(C)** Heatmap presenting the expression levels of the 4 prognostic ERGs. **(D-F)** Kaplan-Meier curves demonstrating the association between the prognostic model’s risk scores and overall survival (OS) in the training and testing cohorts, with higher risk scores correlating with lower survival probabilities. **(G-I)** ROC curve analysis assessing the prognostic accuracy of the model, as indicated by AUC values.

A prognostic framework based on ERGs was constructed to predict patient outcomes, utilizing both Cox proportional hazards regression and Lasso-Cox algorithms. The patient data were split into separate training and testing sets for the purpose of refining and validating the prognostic framework. Through lasso regression, illustrated in **Figures 3A, B**, a prognostic model featuring 4 ERGs (ANGPTL6, POMZP3, LAMA4, RELN) was derived, with details provided in **Supplementary Table 6**. The heatmap in **Figure 3C** displays the expression levels of these ERGs. Kaplan-Meier curves validated the model’s strong association with overall survival (OS) in the studied cohorts (p<0.05) (**Figures 3D–F**), associating higher risk scores with lower 5-year survival probabilities. The model’s prognostic accuracy was assessed by the AUC values of ROC curves (**Figures 3G–I**).

### Delineating the central gene in ERGs network

**Figure 4:**
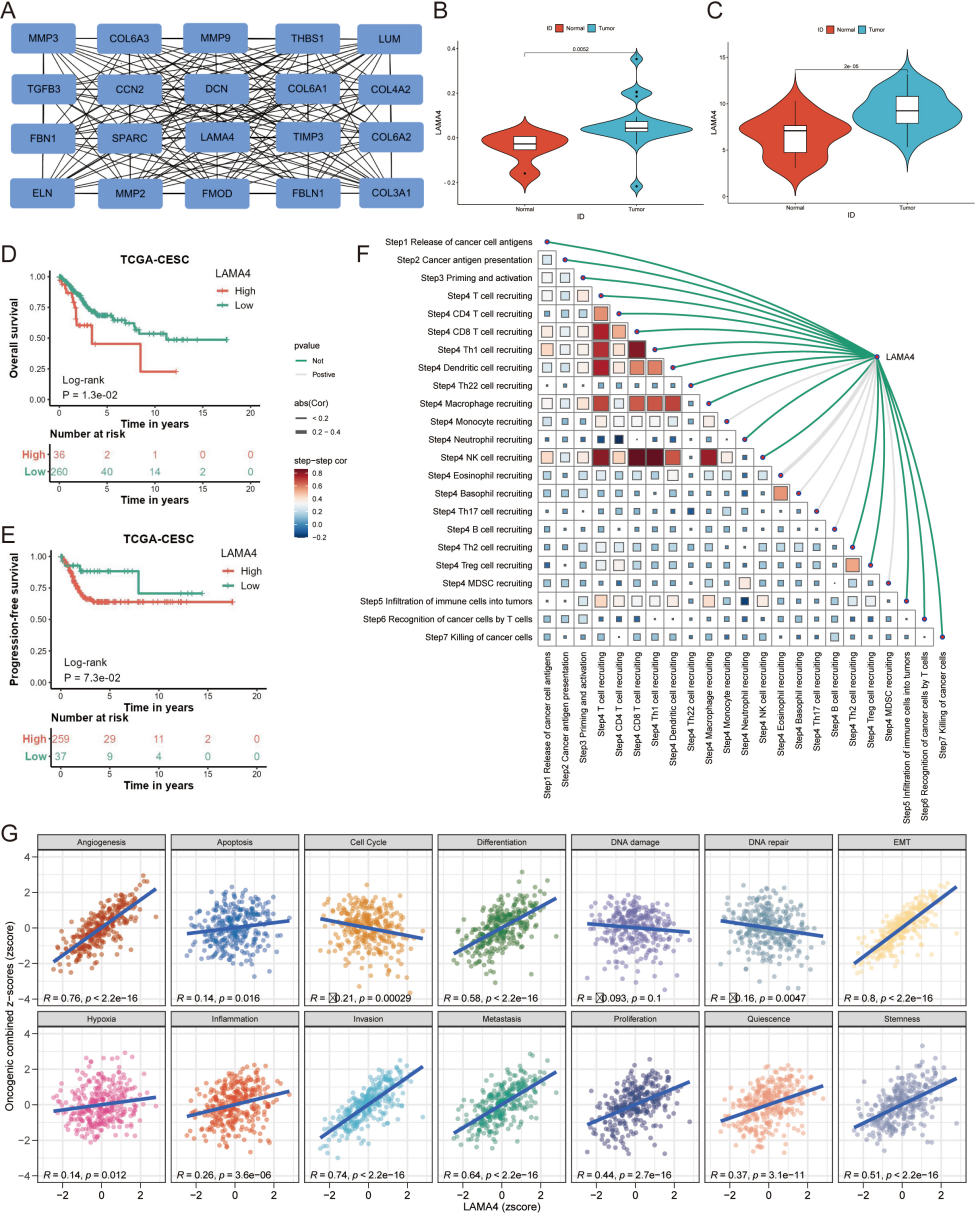
Identification of LAMA4 as a central hub gene and its clinical relevance: **(A)** Network diagram depicting the interactions among ERGs, with key hub genes identified using the MCC algorithm. **(B, C)** Validation of LAMA4 upregulation in cervical cancer tumors compared to adjacent non-tumorous tissues using GSE14404 and GSE63514 datasets. **(D, E)** Kaplan-Meier plots showing the association of LAMA4 expression with overall survival (OS) and progression-free survival (PFS) outcomes. **(F)** Correlation between TIP scoring and LAMA4 expression, indicating the gene’s role in prognostic scoring systems. **(G)** Functional and pathway analyses from the CancerSEA database relating LAMA4 expression to immune cell infiltration and various functional states.

Our investigation into the differentially expressed ERGs in CC led us to identify key or “hub” genes using the MCC algorithm within the cytoHubba plugin (**Figure 4A**). A subset of ERGs—specifically the top 20 with the most pronounced differential expression as gauged by their |logFC| values and those with superior MCC scores within the PPI network—were selected (details in **Supplementary Table 7**). After comparing these candidates with genes from the prognostic model, LAMA4 emerged as the central hub gene, underscored by its highest MCC score.

Analyses revealed that LAMA4 was significantly upregulated in CC tumors relative to adjacent non-tumorous tissues in datasets GSE14404 (p=0.0052) (**Figure 4B**) and GSE63514 (p<0.001) (**Figure 4C**). Such upregulation of LAMA4 was associated with poorer overall survival (OS) and progression-free survival (PFS) outcomes (p<0.001 for both) (**Figures 4D, E**).

**Figure 4F** depicts the correlation between TIP scoring and LAMA4 expression, highlighting the interconnectedness of various prognostic scores. We harnessed a suite of computational analyses to probe the relationship between LAMA4 expression and immune cell infiltration. The CancerSEA database facilitated functional and pathway analyses, revealing notable associations between LAMA4 expression and a range of functional states and pathways (**Figure 4G**).

### Single-cell analysis of LAMA4 expression

**Figure 5:**
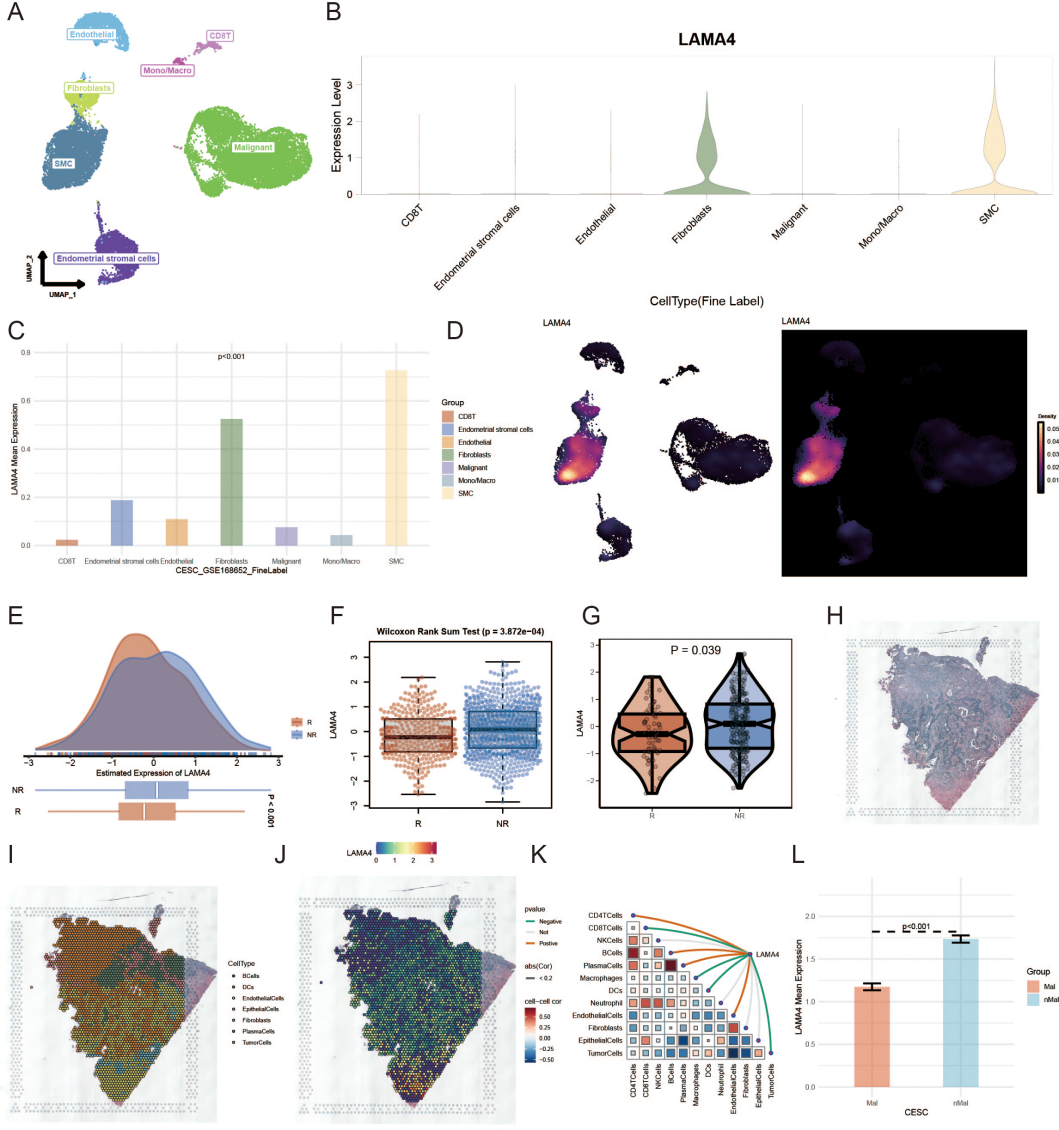
Single-cell and immunotherapy response analysis of LAMA4 in cervical cancer: **(A)** Single-cell RNA sequencing data analysis from GSE168652, exploring LAMA4 expression in the tumor microenvironment (TME). **(B, C)** Elevated expression of LAMA4 in fibroblasts and SMCs, highlighting its potential role in cervical cancer progression. **(D)** UMAP visualization of LAMA4 distribution across different cell types. **(E, F)** Analysis of LAMA4 expression in relation to immunotherapy response, with higher expression observed in non-responders. **(G)** IMvigor210 cohort data supporting the potential of LAMA4 as a predictor of immunotherapy outcomes. **(H-K)** Spatial distribution of LAMA4 in the transcriptome, with an inverse relationship with macrophage expression and confinement to non-tumor areas. **(L)** Spatial expression pattern of LAMA4, demonstrating its significance in the ECM composition.

We explored the expression dynamics of LAMA4 in the tumor microenvironment (TME) by analyzing single-cell RNA sequencing data from GSE168652 (**Figure 5A**). Our analysis highlighted the prominent expression of LAMA4 in fibroblasts and SMCs, suggesting its potential cellular origin in the development of CC (p<0.001) (**Figures 5B, C**). Using UMAP to condense the data into 2D representations, we employed ggplot2 to create visualizations of gene expression distribution among different cell types (**Figure 5D**). It should be noted that this analysis was based on a single-patient dataset (GSE168652), and thus the findings are exploratory and require validation in larger scRNA-seq cohorts.

### LAMA4’s link to immunotherapy outcomes

Our investigation into LAMA4’s expression in relation to a comprehensive immunotherapy gene profile segmented patients into responders (R), including Complete Response (CR) and Partial Response (PR), and non-responders (NR), such as Stable Disease (SD) and Progressive Disease (PD). With an enlarged cohort from the immunotherapy dataset, we noted a higher expression of LAMA4 in the NR groups (**Figures 5E, F**), suggesting that elevated LAMA4 levels may predict a less favorable immunotherapy response, a finding corroborated by data from the IMvigor210 cohort (**Figure 5G**).

### Spatial deconvolution analysis of LAMA4

**Figure 5H** shows the original tissue image. After deconvolution, we pinpointed the cell type most prevalent in each microregion. Using the SpatialDimPlot function from Seurat, we graphically represented the dominant cell populations in each microregion (**Figure 5I**). **Figure 5J** presents the spatial expression pattern of LAMA4, revealing its concentration in specific cell regions and its inverse relationship with macrophage expression. **Figure 5K** further confirms this trend, demonstrating a significant negative association between LAMA4 expression and macrophage abundance in spatial segments. Conversely, **Figure 5L** shows that LAMA4 expression is largely confined to non-tumor areas, indicating its importance in the ECM composition.

### RT-qPCR and IHC

**Figure 6:**
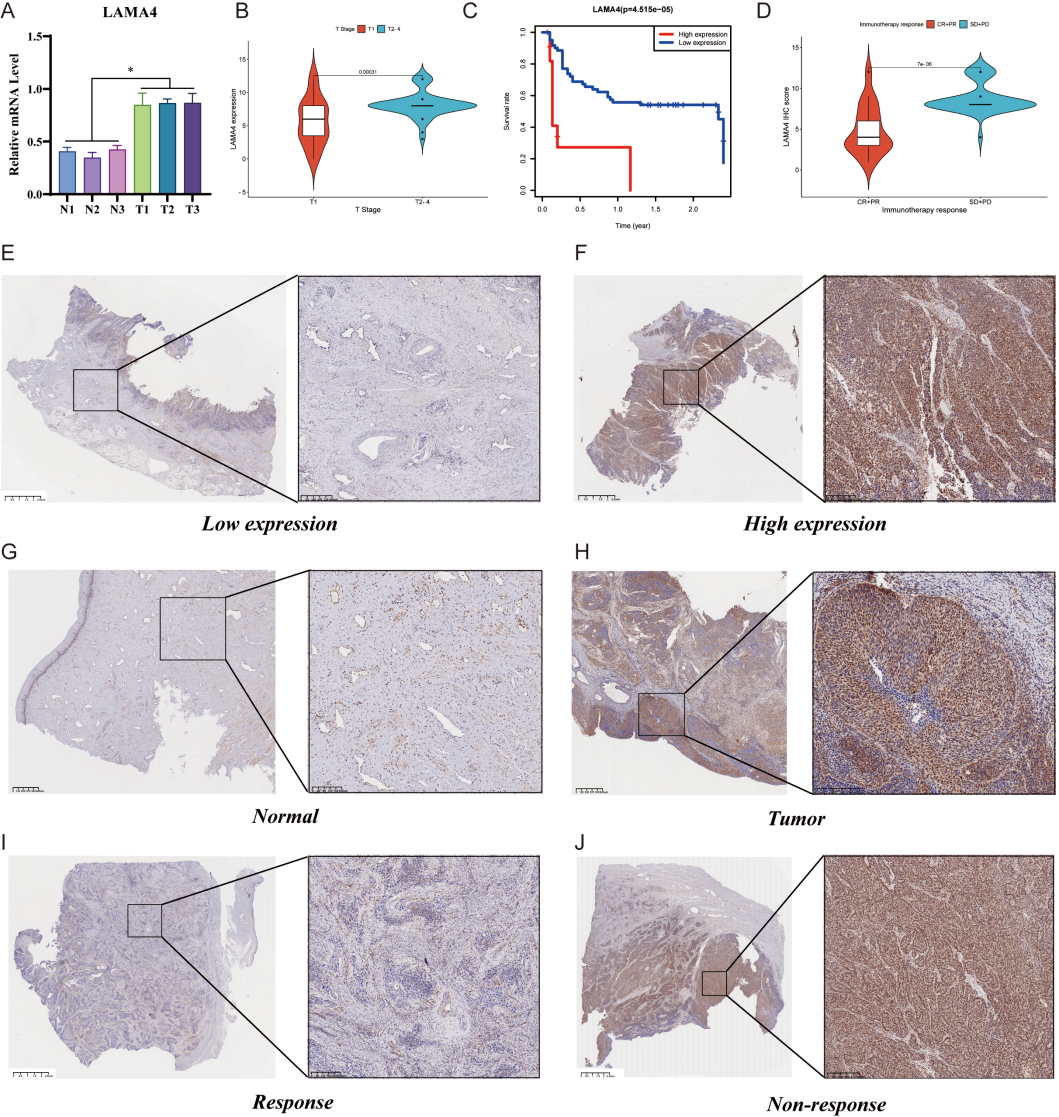
RT-qPCR, IHC analysis of LAMA4, and its prognostic significance: **(A)** RT-qPCR assessment of LAMA4 expression in cervical cancer tissues versus adjacent non-malignant tissues, indicating increased levels in cancerous cells. **(B)** Immunohistochemical analysis of LAMA4 protein abundance in a cohort of 73 cervical cancer patients, with a significant overexpression observed in the majority. **(C)** Kaplan-Meier survival plots stratifying patients by LAMA4 expression levels, showing reduced overall survival (OS) rates associated with higher LAMA4 levels. **(D)** Correlation of LAMA4 protein expression with the efficacy of immunotherapy in patients receiving ICI therapy, with higher expression linked to less favorable immediate outcomes. **(E-H)** Variations in LAMA4 expression levels across different patient samples. **(I-J)** Further validation of LAMA4 as an indicator for predicting immunotherapy response in cervical cancer patients.

Using RT-qPCR, we measured the expression of LAMA4 in CC tissues in comparison to adjacent non-malignant tissues. The data indicated a significant increase in LAMA4 levels in the CC specimens relative to the healthy tissue, pointing to its heightened expression in the cancerous cells (**Figure 6A**).

The protein abundance of LAMA4 was evaluated in 73 CC patients using immunohistochemistry (IHC), with an unusual overexpression noted in 68.5% (50/73) of the participants. The link between LAMA4 levels and a range of clinicopathological parameters was examined, with the specifics presented in **Table 1**. Remarkably, there was no significant relationship between the expression of LAMA4 and the patients’ age, sex, or the N and M classifications. However, LAMA4 was found to be considerably more expressed in CC tissues at stages T2–4 than at stage T1 (**Figure 6B**).

After categorizing the patients into groups with either high or low LAMA4 expression based on the median values, Kaplan-Meier survival plots showed that those with higher levels of LAMA4 had a reduced overall survival (OS) rate (p=0.00031), highlighting its role as a prospective prognostic marker for CC (**Figure 6C**). **Figures 6E–H** display the variations in LAMA4 expression levels.

### Correlation between LAMA4 expression and immunotherapy efficacy

In terms of the association between LAMA4 expression and the efficacy of immunotherapy, we examined the LAMA4 protein expression in 55 patients receiving ICI therapy for advanced or metastatic CC. This group included 2 complete responses (CR), 15 partial responses (PR), 37 stable diseases (SD), and 1 progressive disease (PD). We associated the IHC scores with the effectiveness of the therapy and noted that the SD+PD category had increased LAMA4 IHC scores in comparison to the CR+PR category (**Figure 6D**). These findings are in agreement with data from other immunotherapy cohorts and imply that patients with higher LAMA4 levels might have less favorable immediate outcomes after ICI treatment, making LAMA4 a promising indicator for anticipating the response to treatment in CC (**Figures 6I, J**).

## Discussion

An increasing body of research has revealed that the interactions between the tumor microenvironment (TME) and tumor cells are crucial for the progression and metastasis of malignancies (12). Tumor cells can alter and maintain local conditions favorable to their survival and development through various mechanisms, including protein secretion; conversely, the TME can promote the invasive and metastatic capabilities of tumor cells by influencing their biological functions (13, 14). The TME primarily consists of the ECM, soluble molecules, and stromal cells, with the ECM serving as a key structural framework that regulates vital activities such as tumor cell growth, invasion, apoptosis, drug resistance, and metastasis, making it an essential condition for tumor cell survival (15). The loss of ECM stability is considered one of the key factors in the development and metastasis of malignant tumor cells (16).

TME is not merely a passive scaffold but a dynamic and integral component of cancer progression. Central to the TME is the ECM, which undergoes extensive remodeling during tumorigenesis. This process, characterized by a shift in the balance between ECM protein synthesis and degradation by enzymes like matrix metalloproteinases (MMPs), fundamentally alters the tissue architecture. In cervical cancer, this pathological remodeling disrupts the basement membrane, facilitating invasion and metastasis. Furthermore, the remodeled ECM creates a pro-tumorigenic niche by altering mechanical properties (e.g., tissue stiffness), releasing sequestered growth factors, and presenting new signaling cues to cancer cells. Therefore, the ECM plays a pivotal role in the growth and invasion of CC cells. Current research has delved into the interplay between ECM-related proteins and signaling pathways and found a close association between the ECM and the effectiveness of tumor immunotherapy, with some ECM-related molecules already being utilized for clinical diagnosis and treatment (17, 18). The findings of this study provide a substantial contribution to the current understanding of ERGs and their prognostic significance in CC. Our research has uncovered a novel prognostic signature based on ERGs, with LAMA4 identified as a central gene associated with both prognosis and response to immunotherapy in CC patients.

A key strength of our study is the successful categorization of CC patients into two distinct molecular subtypes via consensus clustering based on ERG expression. The subtype associated with poorer survival (Cluster A) was significantly enriched in pathways critical to malignancy, such as KEGG_FOCAL_ADHESION, KEGG_ECM_RECEPTOR_INTERACTION, and KEGG_PATHWAYS_IN_CANCER. This finding is highly significant as it suggests that the aggressive phenotype in a subset of CC patients is driven by a distinct, ECM-activated biological program. This molecular stratification provides a strong rationale for the prognostic power of our ERG-based model and underscores that the ECM is not merely a structural scaffold but an active signaling hub that dictates tumor behavior and patient fate. Such subtyping could hold future promise for patient stratification in clinical trials targeting ECM-related pathways.

LAMA4, a member of the laminin gene family (19), was found to be significantly overexpressed in CC tissues compared to adjacent non-cancerous tissues. This overexpression was linked to worse overall survival and progression-free survival, which is consistent with the role of laminins in tumor invasion and metastasis. The prognostic model developed in this study, which includes LAMA4 among other ERGs, has shown robust predictive power for patient outcomes. This suggests that the interplay of these genes could be crucial in the progression of CC and may serve as a reliable predictor of patient prognosis.

Perhaps our most intriguing and unexpected finding was the strong nuclear localization of LAMA4 observed in our IHC analysis. Traditionally, LAMA4 is recognized for its canonical function as a structural protein within the basement membrane. However, its presence in the nucleus strongly suggests a non-canonical, or moonlighting function. It is plausible that LAMA4, or more likely a proteolytically cleaved fragment generated during ECM remodeling, translocates to the nucleus. There, it could act as a transcriptional co-regulator or participate in other nuclear processes, a phenomenon previously reported for other ECM-related proteins like perlecan and endorepellin (20–23). This discovery challenges the conventional view of LAMA4’s role being confined to the extracellular space. It implies that LAMA4 may exert its pro-tumorigenic effects through a two-pronged mechanism: externally, by contributing to the structural and signaling landscape of the remodeled ECM, and internally, by directly influencing gene expression programs within the cancer cell nucleus.

Recent advancements in immunotherapy strategies centered on PD-L1/PD-1 immune checkpoints have brought new hope to CC treatment (24). However, the overall response rates to PD-L1/PD-1 checkpoint inhibitors among CC patients remain modest, revealing a considerable subset of patients for whom these treatments are ineffective (25). Additionally, the research on biomarkers specific to CC is still inadequate, posing key challenges in the field of CC treatment. Hence, immunotherapy research in CC, particularly in identifying biomarkers capable of predicting diagnosis, metastasis, recurrence, and treatment response, has become a focal point of current research. With the progression of high-throughput sequencing technologies, numerous emerging biomarkers have been identified and closely linked to cancer patient diagnosis and prognosis. The exploration and validation of novel, reliable biomarkers are of paramount clinical importance for enhancing early detection rates of CC, improving prognostic assessments, and guiding personalized treatment strategies. Research in this field not only holds the promise of optimizing existing treatment strategies but may also offer more precise, individualized treatment options for CC patients.

The significant association between high LAMA4 expression and poor response to immunotherapy is particularly noteworthy. Our data indicate that patients with elevated LAMA4 levels may not benefit as much from ICI therapy as those with lower levels. The biological underpinnings for this observation are likely multifactorial. Firstly, LAMA4, as a core component of the laminin family, contributes to the structural integrity of the ECM (26). Its overexpression can lead to a denser, more rigid ECM, creating a physical barrier that hinders the infiltration of cytotoxic T-cells into the tumor core, a primary mechanism of immune exclusion (27). Secondly, LAMA4’s interaction with cell surface receptors like integrins can activate intracellular signaling pathways (e.g., FAK, PI3K/AKT) that promote cell survival, proliferation, and resistance to apoptosis, thereby counteracting the effects of immune-mediated cell killing (28). This establishes LAMA4 not just as a biomarker, but as a potential functional driver of an immunosuppressive TME.

Our initial investigation is subject to certain constraints. The limited number of cases included from the hospital’s cohort may affect the statistical strength and the generalizability of our conclusions. We are currently addressing this by enlarging our cohort and intend to integrate additional patient data in future research to bolster the credibility and representativeness of our findings. At the same time, we acknowledge that the scRNA-seq and spatial deconvolution analysis are based on a single sample, which is a major limitation. Additionally, while the bioinformatics analysis of publicly accessible datasets has yielded important preliminary insights, experimental confirmation is essential to substantiate LAMA4’s involvement in CC progression. Our forthcoming studies will engage both *in vitro* and *in vivo* models to authenticate the influence of LAMA4 on pivotal signaling pathways, as well as its contribution to cancer development and response to therapy.

## Conclusion

In conclusion, our study presents compelling evidence that LAMA4, along with other ERGs, could serve as a valuable prognostic tool and a predictor of immunotherapy response in CC. These findings open avenues for further research to explore the mechanistic underpinnings of LAMA4’s role in CC and its potential as a therapeutic target.

## Data Availability

The original contributions presented in the study are included in the article/**Supplementary Material**. Further inquiries can be directed to the corresponding authors.
