# Supplementary material for: Extracellular matrix-related genes-based prognostic signature for cervical cancer: association of LAMA4 expression with prognosis and response to immunotherapy
Source: Front Oncol. 2025 Aug 13;15:1562115. doi: 10.3389/fonc.2025.1562115 (PMC12380540; doi:10.3389/fonc.2025.1562115)
Supplement: Supplementary file 4 [file Table4.docx]

**Supplementary Table 1:** The list of extracellular matrix-related genes (ERGs) from relevant literature.

| A1BG | CDON | ELFN2 | LAMA1 | NDNF | SPON1 |
| --- | --- | --- | --- | --- | --- |
| A2M | CFP | ELN | LAMA2 | NDP | SPON2 |
| ABI3BP | CHAD | EMID1 | LAMA3 | NID1 | SPP2 |
| ACAN | CHADL | EMILIN1 | LAMA4 | NID2 | SRPX |
| ACHE | CHI3L1 | EMILIN2 | LAMA5 | NPNT | SRPX2 |
| ADAM11 | CILP | EMILIN3 | LAMB1 | NPPA | SSC5D |
| ADAM19 | CLC | ENAM | LAMB2 | NTN1 | SSPOP |
| ADAMDEC1 | CLEC14A | ENSG00000278817 | LAMB3 | NTN3 | SULF1 |
| ADAMTS1 | CLEC3B | ENTPD2 | LAMB4 | NTN4 | TECTA |
| ADAMTS10 | CLU | EPYC | LAMC1 | NTN5 | TECTB |
| ADAMTS12 | CMA1 | ERBIN | LAMC2 | NTNG1 | TFIP11 |
| ADAMTS13 | COCH | EYS | LAMC3 | NTNG2 | TFPI2 |
| ADAMTS14 | COL10A1 | F12 | LEFTY2 | NYX | TGFB1 |
| ADAMTS15 | COL11A1 | F13A1 | LGALS1 | OC90 | TGFB1I1 |
| ADAMTS16 | COL11A2 | F2 | LGALS3 | OGN | TGFB2 |
| ADAMTS17 | COL12A1 | F3 | LGALS3BP | OLFML2A | TGFB3 |
| ADAMTS18 | COL13A1 | F7 | LGALS4 | OMD | TGFBI |
| ADAMTS19 | COL14A1 | F9 | LGALS9 | OPTC | TGFBR3 |
| ADAMTS2 | COL15A1 | FBLN1 | LINGO1 | ORM1 | TGM2 |
| ADAMTS20 | COL16A1 | FBLN2 | LINGO2 | ORM2 | TGM4 |
| ADAMTS3 | COL17A1 | FBLN5 | LINGO3 | OTOG | THBS1 |
| ADAMTS4 | COL18A1 | FBN1 | LINGO4 | OTOGL | THBS2 |
| ADAMTS5 | COL19A1 | FBN2 | LMAN1 | OTOL1 | THBS3 |
| ADAMTS6 | COL1A1 | FBN3 | LMAN1L | P3H1 | THBS4 |
| ADAMTS7 | COL1A2 | FCGBP | LOX | P3H2 | THSD4 |
| ADAMTS8 | COL20A1 | FCN1 | LOXL1 | PAPLN | TIMP1 |
| ADAMTS9 | COL21A1 | FCN2 | LOXL2 | PCOLCE | TIMP2 |
| ADAMTSL1 | COL22A1 | FCN3 | LRIG1 | PCSK6 | TIMP3 |
| ADAMTSL2 | COL23A1 | FGA | LRIG2 | PDGFB | TIMP4 |
| ADAMTSL3 | COL24A1 | FGB | LRIG3 | PF4 | TINAG |
| ADAMTSL4 | COL25A1 | FGF1 | LRRC15 | PHOSPHO1 | TINAGL1 |
| ADAMTSL5 | COL26A1 | FGF10 | LRRC17 | PI3 | TLR3 |
| ADIPOQ | COL27A1 | FGF9 | LRRC24 | PKM | TMEFF1 |
| AEBP1 | COL28A1 | FGFBP3 | LRRC32 | PLG | TMEFF2 |
| AGRN | COL2A1 | FGFR2 | LRRC3B | PLOD3 | TNC |
| AGT | COL3A1 | FGG | LRRC3C | PLSCR1 | TNFRSF11B |
| AHSG | COL4A1 | FGL1 | LRRN1 | PODN | TNN |
| ALPL | COL4A2 | FGL2 | LRRN2 | PODNL1 | TNR |
| AMBP | COL4A3 | FIBCD1 | LRRN3 | POMZP3 | TNXB |
| AMELX | COL4A4 | FLG | LRRTM1 | POSTN | TPSAB1 |
| AMELY | COL4A5 | FLRT1 | LRRTM3 | PRELP | TPSB2 |
| AMTN | COL4A6 | FLRT2 | LRRTM4 | PRG2 | TRIL |
| ANG | COL5A1 | FLRT3 | LTBP1 | PRG3 | UCMA |
| ANGPT1 | COL5A2 | FMOD | LTBP2 | PRG4 | USH2A |
| ANGPT2 | COL5A3 | FN1 | LTBP3 | PRSS1 | VASN |
| ANGPT4 | COL6A1 | FRAS1 | LTBP4 | PRSS2 | VCAN |
| ANGPTL1 | COL6A2 | FREM1 | LUM | PRTN3 | VEGFA |
| ANGPTL2 | COL6A3 | FREM2 | MARCOL | PSAP | VIT |
| ANGPTL3 | COL6A5 | FREM3 | MATN1 | PTN | VTN |
| ANGPTL4 | COL6A6 | GDF10 | MATN2 | PTPRZ1 | VWA1 |
| ANGPTL5 | COL7A1 | GDF15 | MATN3 | PTX3 | VWA2 |
| ANGPTL6 | COL8A1 | GFOD2 | MATN4 | PXDN | VWC2 |
| ANGPTL7 | COL8A2 | GH1 | MBL2 | PZP | VWF |
| ANOS1 | COL9A1 | GLG1 | MDK | RARRES2 | WNT11 |
| ANXA1 | COL9A2 | GP1BA | MEGF9 | RBP3 | WNT2 |
| ANXA11 | COL9A3 | GPC1 | MEPE | RELL2 | WNT2B |
| ANXA2 | COLEC12 | GPC2 | MFAP1 | RELN | WNT3 |
| ANXA2P2 | COLQ | GPC3 | MFAP2 | RTBDN | WNT4 |
| ANXA4 | COMP | GPC4 | MFAP4 | RTN4RL1 | WNT5A |
| ANXA5 | CPA3 | GPC5 | MFAP5 | RTN4RL2 | WNT5B |
| ANXA6 | CPN2 | GPC6 | MFGE8 | S100A10 | WNT6 |
| ANXA7 | CRELD1 | GPLD1 | MGP | S100A4 | WNT7A |
| ANXA8 | CRISP3 | GREM1 | MMP1 | S100A6 | WNT8A |
| APCS | CRISPLD2 | HAPLN1 | MMP10 | S100A7 | ZAN |
| APLP1 | CSPG4 | HAPLN2 | MMP11 | S100A8 | ZG16 |
| APOA1 | CST3 | HAPLN3 | MMP12 | S100A9 | ZP1 |
| APOA4 | CSTB | HAPLN4 | MMP13 | SBSPON | ZP2 |
| APOC3 | CTHRC1 | HDGF | MMP14 | SCARA3 | ZP3 |
| APOE | CTSB | HMCN1 | MMP15 | SDC2 |  |
| APOH | CTSC | HMCN2 | MMP16 | SDC3 |  |
| ASPN | CTSD | HNRNPM | MMP17 | SEMA3B |  |
| ATRN | CTSF | HPSE | MMP19 | SEMA7A |  |
| ATRNL1 | CTSG | HPSE2 | MMP2 | SERAC1 |  |
| AZGP1 | CTSH | HPX | MMP20 | SERPINA1 |  |
| BCAM | CTSL | HRG | MMP21 | SERPINA3 |  |
| BCAN | CTSS | HRNR | MMP23B | SERPINA5 |  |
| BGN | CTSZ | HSD17B12 | MMP24 | SERPINB1 |  |
| BMP7 | CXCL12 | HSP90B1 | MMP25 | SERPINB12 |  |
| BMPER | DAG1 | HSPG2 | MMP26 | SERPINB6 |  |
| C17orf58 | DCN | HTRA1 | MMP27 | SERPINB8 |  |
| C1QA | DEFA1 | ICAM1 | MMP28 | SERPINB9 |  |
| C1QB | DEFA1B | IFNA2 | MMP3 | SERPINC1 |  |
| C1QC | DGCR6 | IGFALS | MMP7 | SERPINE1 |  |
| CALR | DLG1 | IGFBP7 | MMP8 | SERPINE2 |  |
| CASK | DMBT1 | IHH | MMP9 | SERPINF1 |  |
| CBLN1 | DMP1 | IL7 | MMRN1 | SERPINF2 |  |
| CBLN4 | DPT | IMPG1 | MMRN2 | SERPING1 |  |
| CCBE1 | DSPP | IMPG2 | MST1 | SERPINH1 |  |
| CCDC80 | DST | INHBE | MUC17 | SFRP1 |  |
| CCN1 | ECM1 | ITGA6 | MUC2 | SFRP2 |  |
| CCN2 | ECM2 | ITIH1 | MUC4 | SHH |  |
| CCN3 | EDIL3 | ITIH2 | MUC5AC | SLPI |  |
| CCN4 | EFEMP1 | ITIH4 | MUC5B | SMOC1 |  |
| CCN5 | EFEMP2 | ITIH5 | MUC6 | SMOC2 |  |
| CCN6 | EFNA5 | KAZALD1 | MXRA5 | SNORC |  |
| CD151 | EGFL6 | KERA | MXRA7 | SOD3 |  |
| CD180 | EGFL7 | KNG1 | MYOC | SOST |  |
| CD248 | EGFLAM | KRT1 | NAV2 | SPARC |  |
| CDH13 | ELANE | L1CAM | NCAM1 | SPARCL1 |  |
| CDH2 | ELFN1 | LAD1 | NCAN | SPOCK2 |  |
