# Supplementary figures and images for: Extracellular matrix-related genes-based prognostic signature for cervical cancer: association of LAMA4 expression with prognosis and response to immunotherapy
Source: Front Oncol. 2025 Aug 13;15:1562115. doi: 10.3389/fonc.2025.1562115 (PMC12380540; doi:10.3389/fonc.2025.1562115)

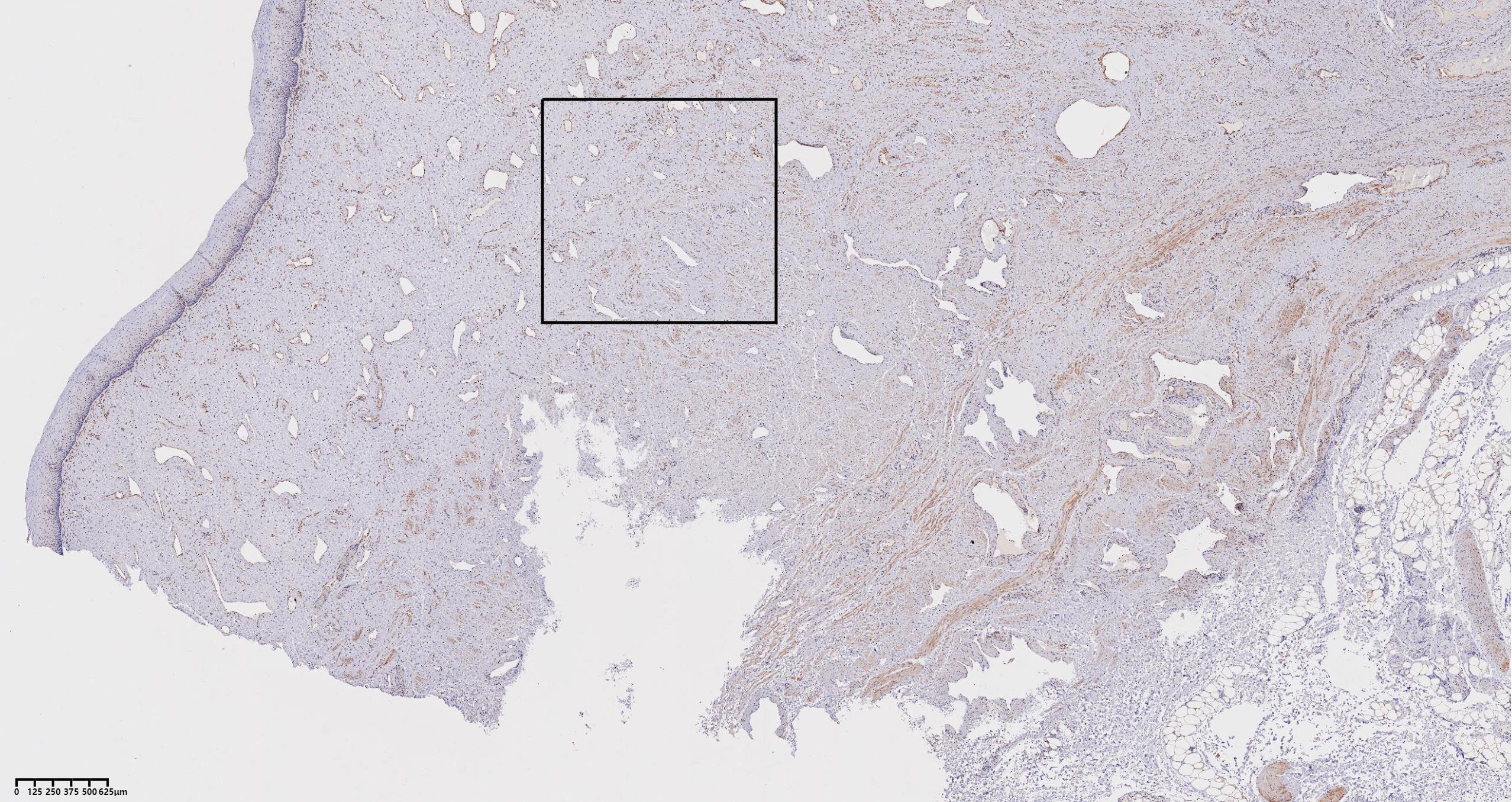

Supplement: Supplementary file 8 [file DataSheet1.zip › original data/3.qPCR+IHC+Clinical data from our hospital/2IHC (Due to ethical requirements, only partial data can be presented)/C10WH LAMA4_2.42X.jpg]

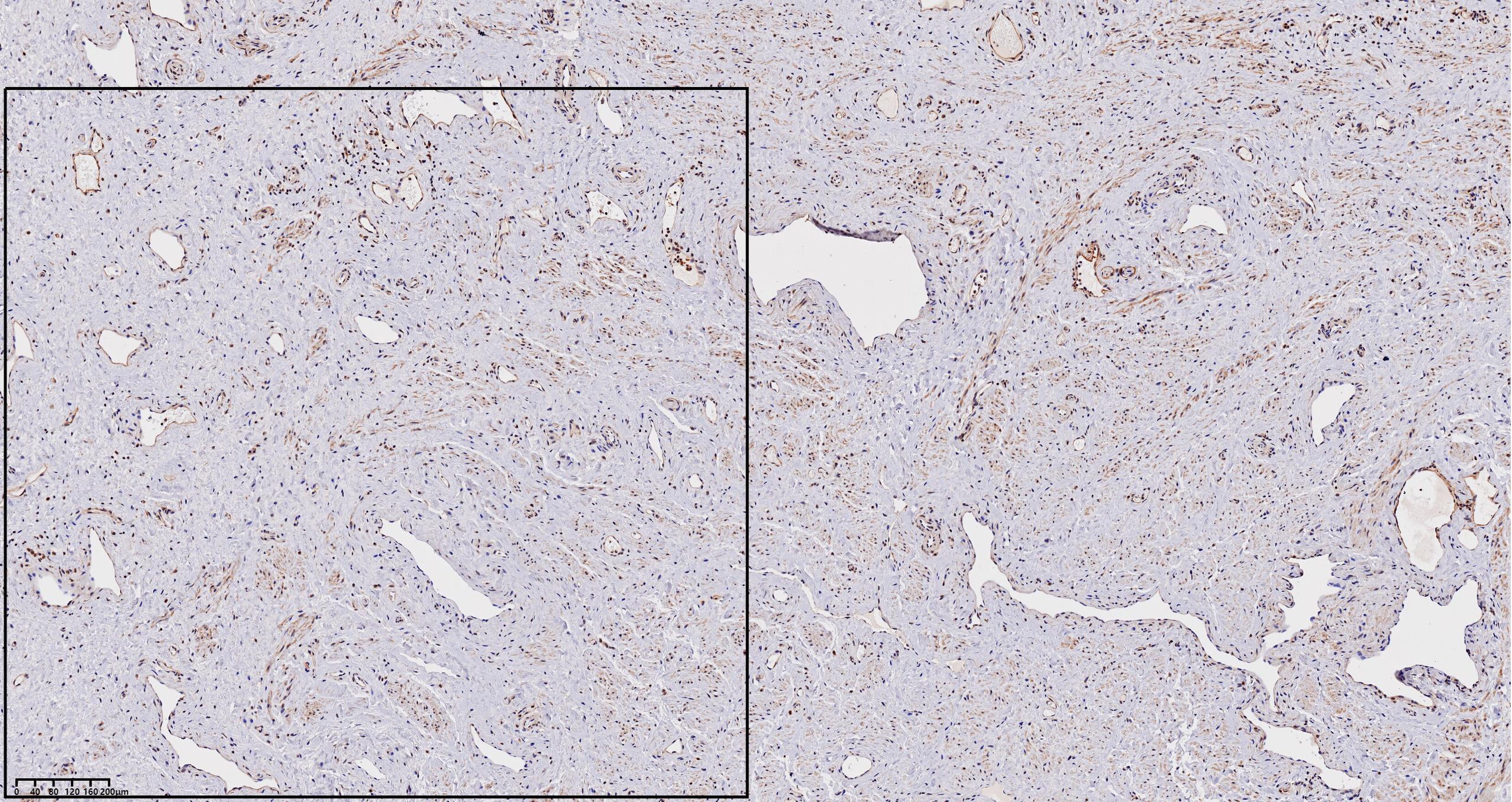

Supplement: Supplementary file 8 [file DataSheet1.zip › original data/3.qPCR+IHC+Clinical data from our hospital/2IHC (Due to ethical requirements, only partial data can be presented)/C10WH LAMA4_7.70X.jpg]

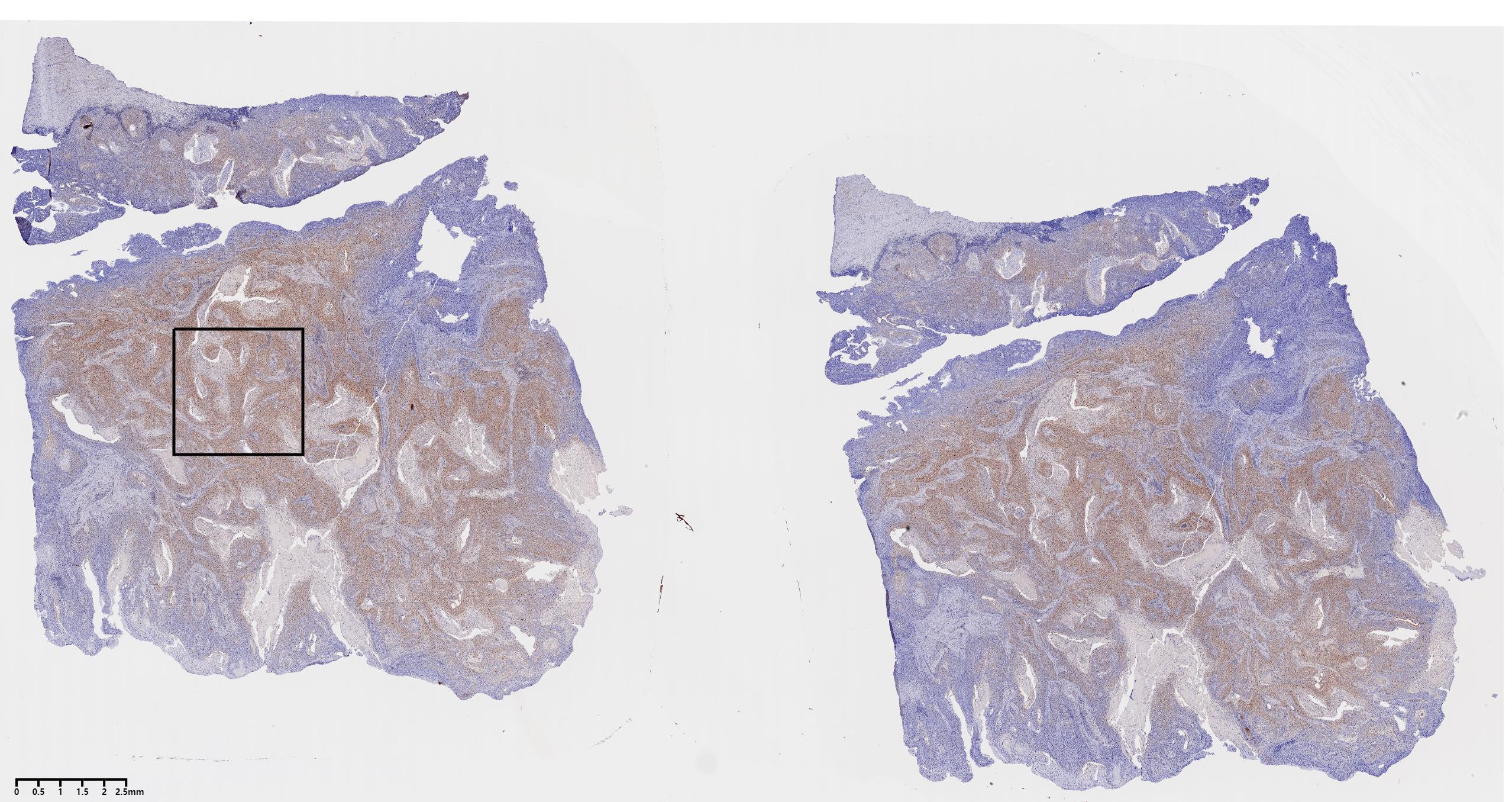

Supplement: Supplementary file 8 [file DataSheet1.zip › original data/3.qPCR+IHC+Clinical data from our hospital/2IHC (Due to ethical requirements, only partial data can be presented)/C11ZWH LAMA4_0.73X.jpg]

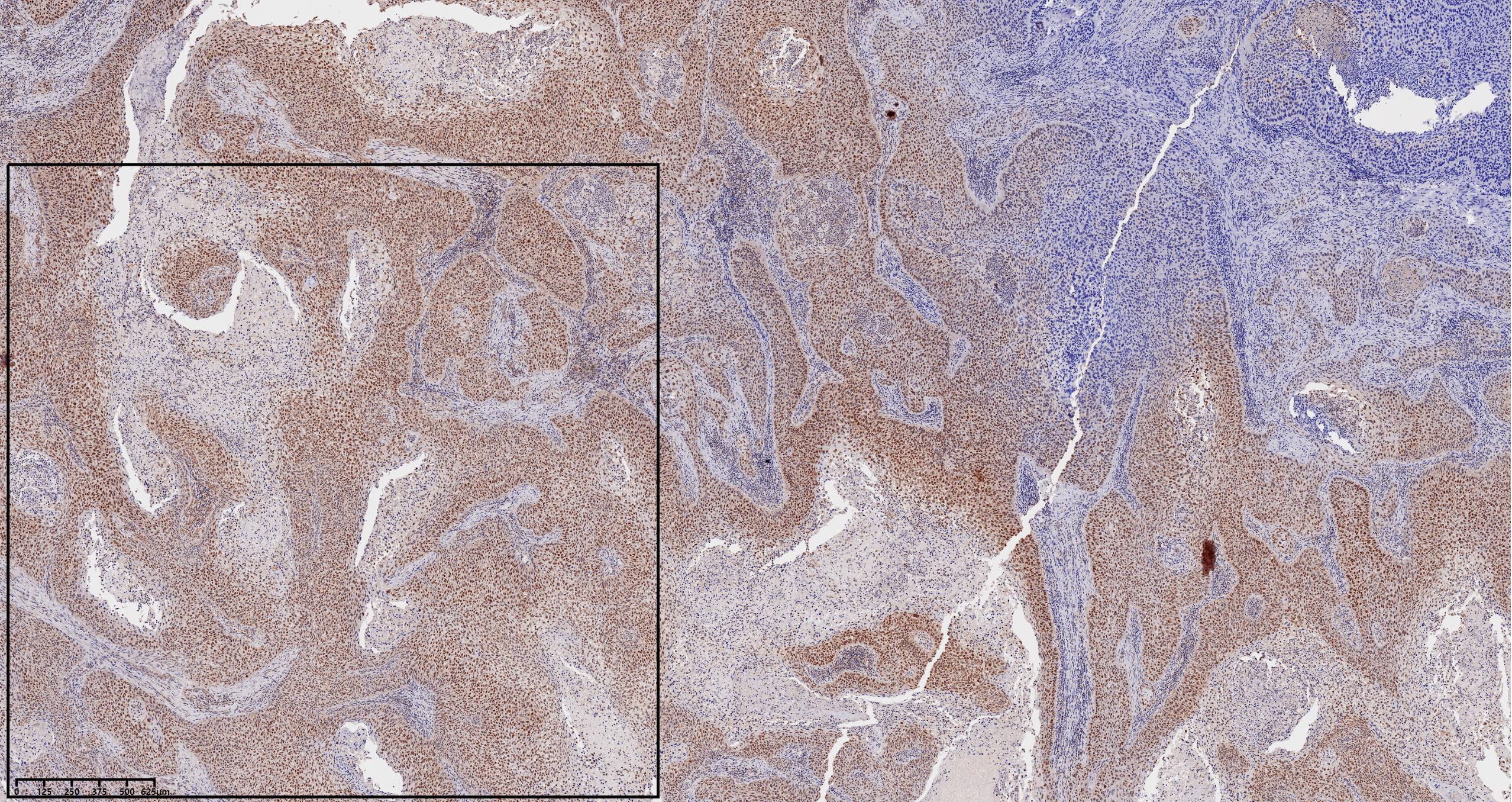

Supplement: Supplementary file 8 [file DataSheet1.zip › original data/3.qPCR+IHC+Clinical data from our hospital/2IHC (Due to ethical requirements, only partial data can be presented)/C11ZWH LAMA4_3.67X.jpg]

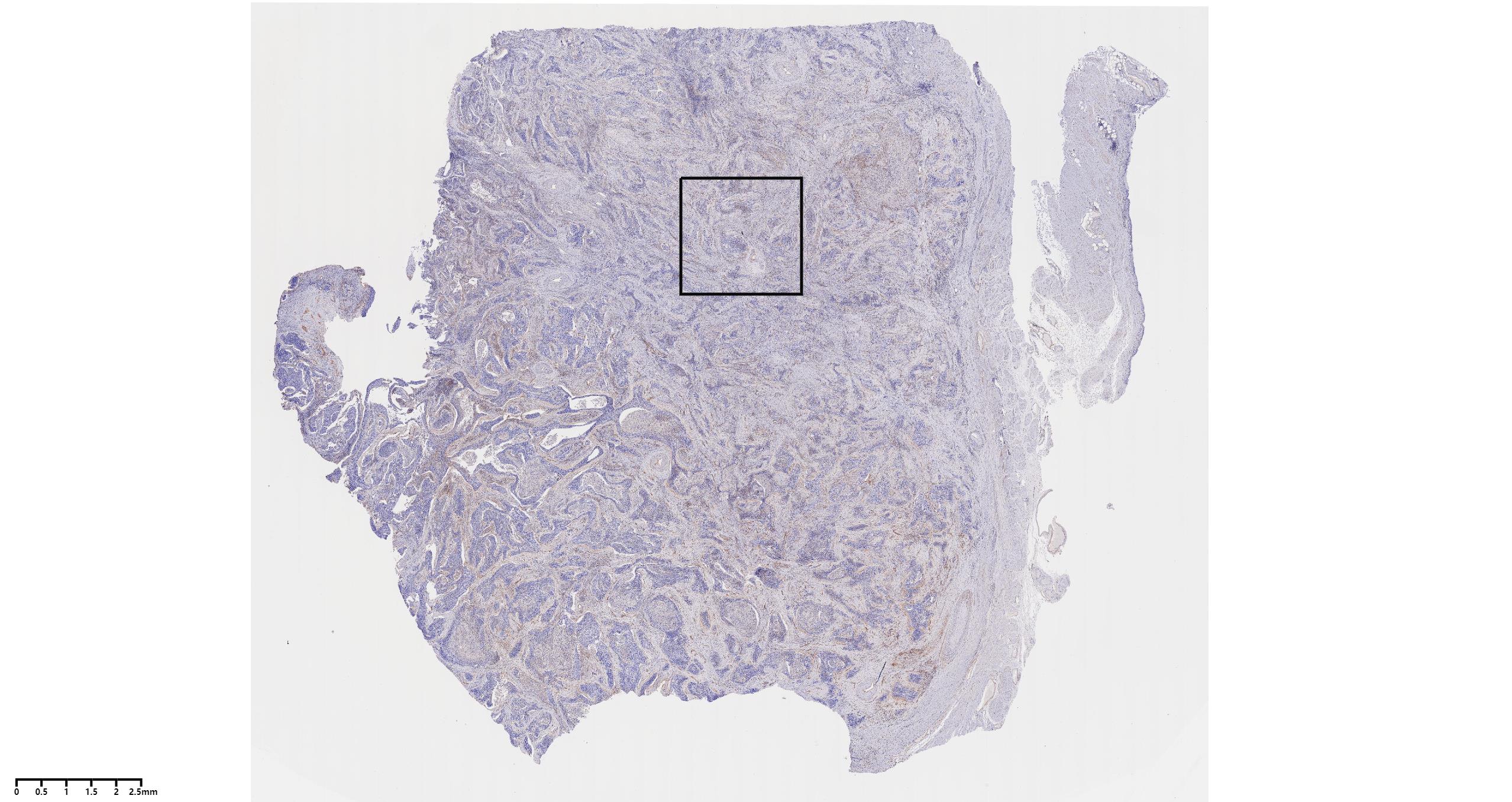

Supplement: Supplementary file 8 [file DataSheet1.zip › original data/3.qPCR+IHC+Clinical data from our hospital/2IHC (Due to ethical requirements, only partial data can be presented)/C1ZXL LAMA4_0.83X-02.jpg]

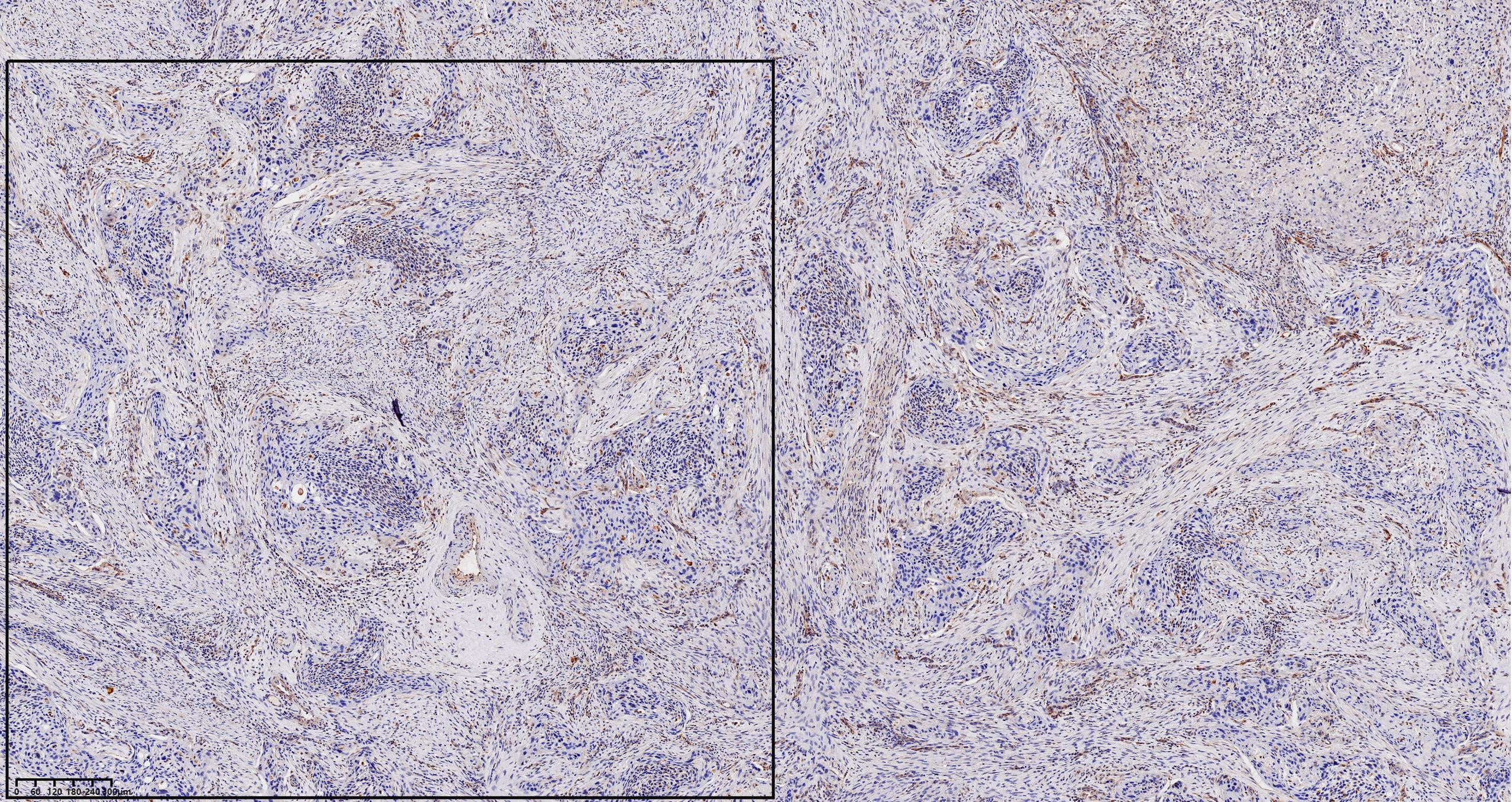

Supplement: Supplementary file 8 [file DataSheet1.zip › original data/3.qPCR+IHC+Clinical data from our hospital/2IHC (Due to ethical requirements, only partial data can be presented)/C1ZXL LAMA4_5.27X.jpg]

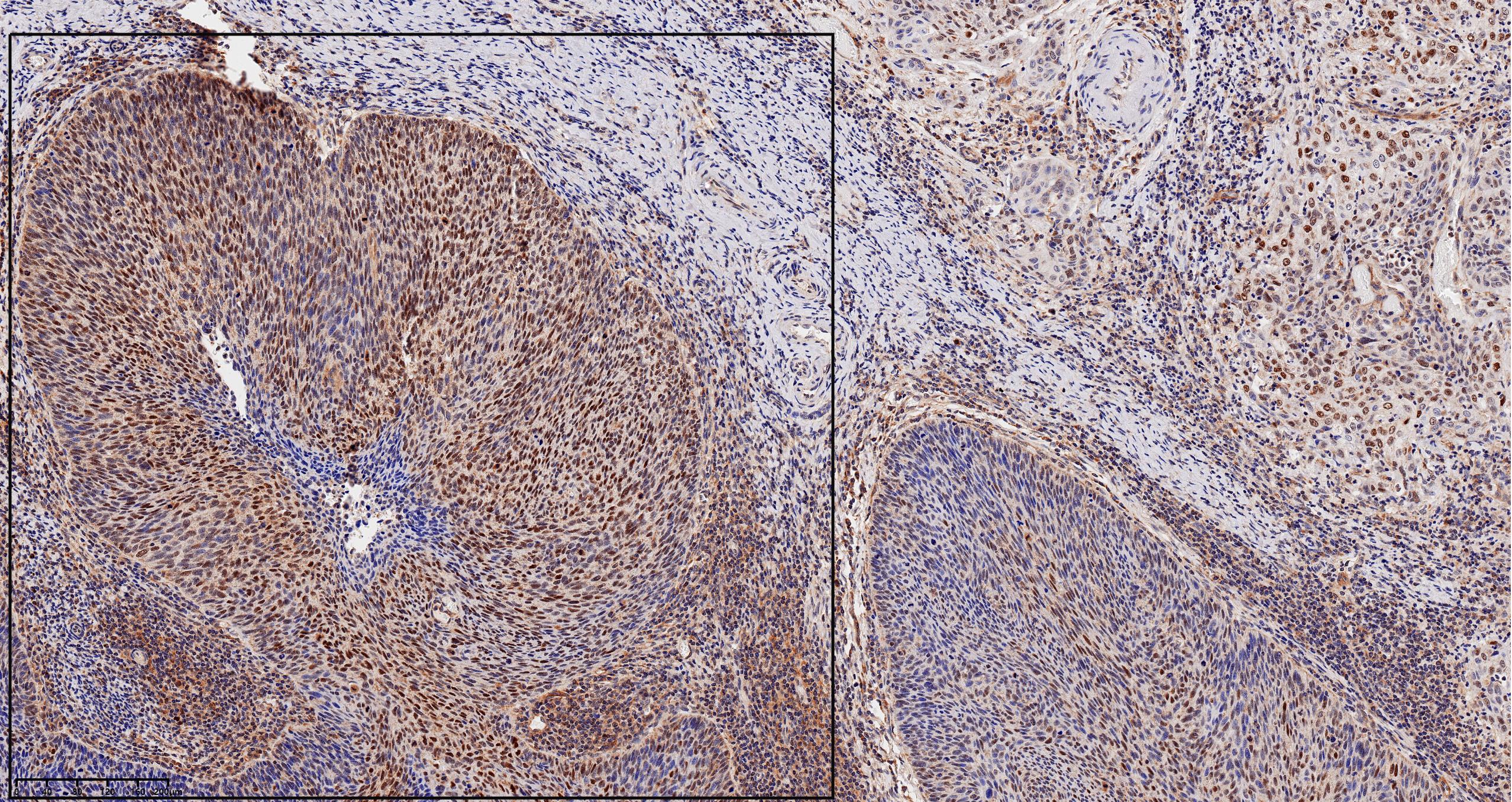

Supplement: Supplementary file 8 [file DataSheet1.zip › original data/3.qPCR+IHC+Clinical data from our hospital/2IHC (Due to ethical requirements, only partial data can be presented)/C3ZJH LAMA4_12.61X.jpg]

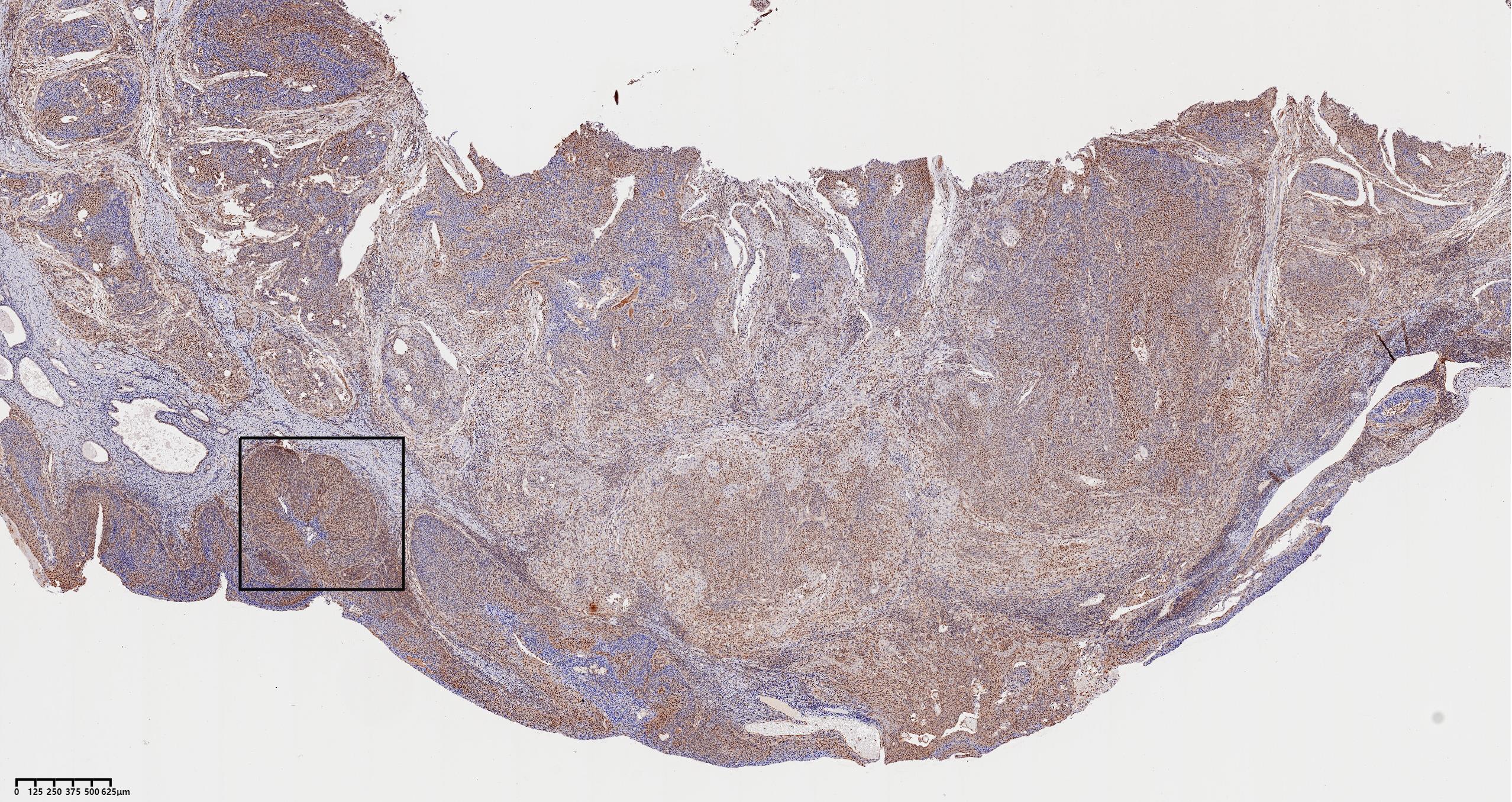

Supplement: Supplementary file 8 [file DataSheet1.zip › original data/3.qPCR+IHC+Clinical data from our hospital/2IHC (Due to ethical requirements, only partial data can be presented)/C3ZJH LAMA4_2.50X.jpg]

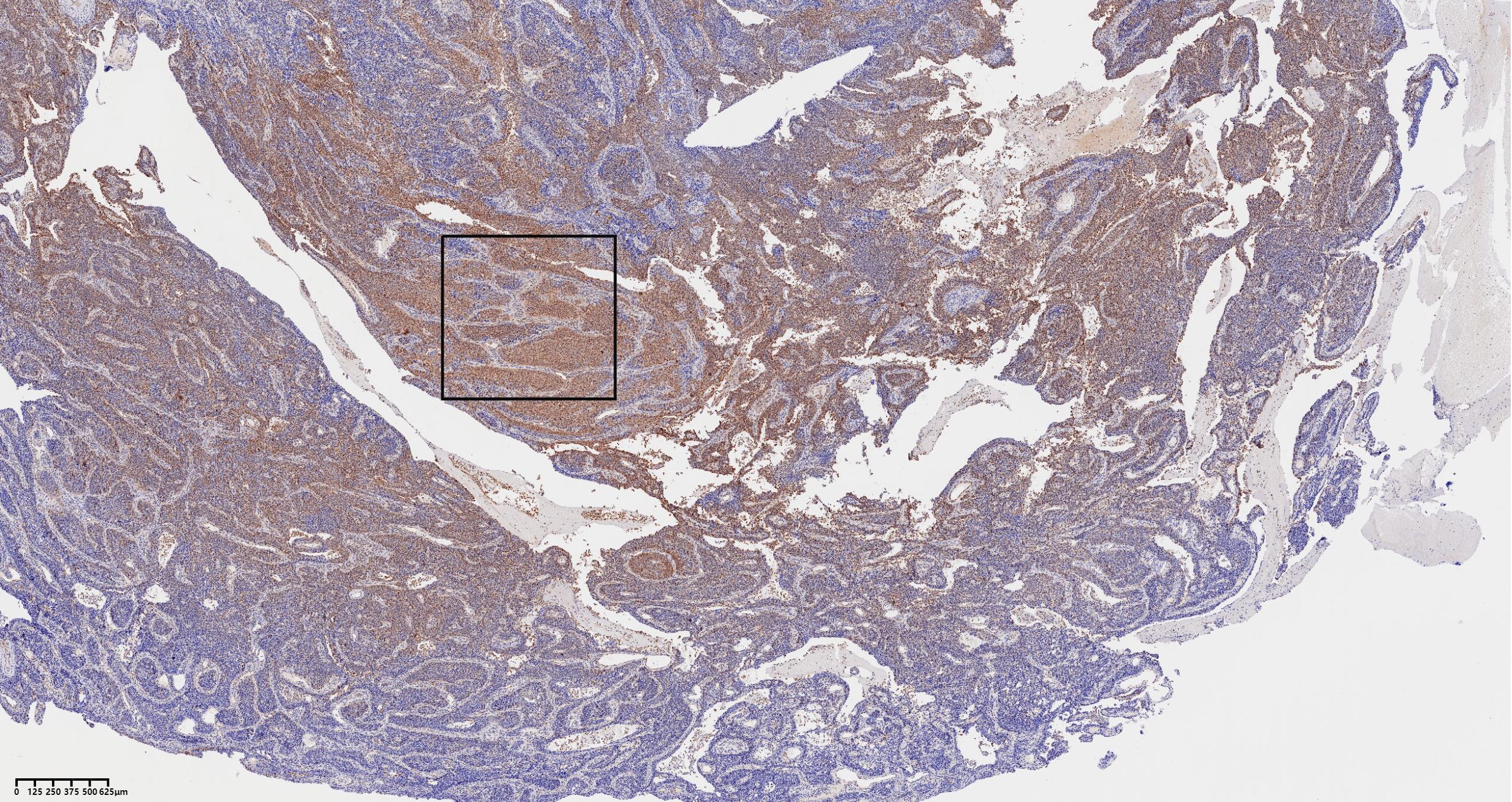

Supplement: Supplementary file 8 [file DataSheet1.zip › original data/3.qPCR+IHC+Clinical data from our hospital/2IHC (Due to ethical requirements, only partial data can be presented)/C4LYL LAMA4_2.44X.jpg]

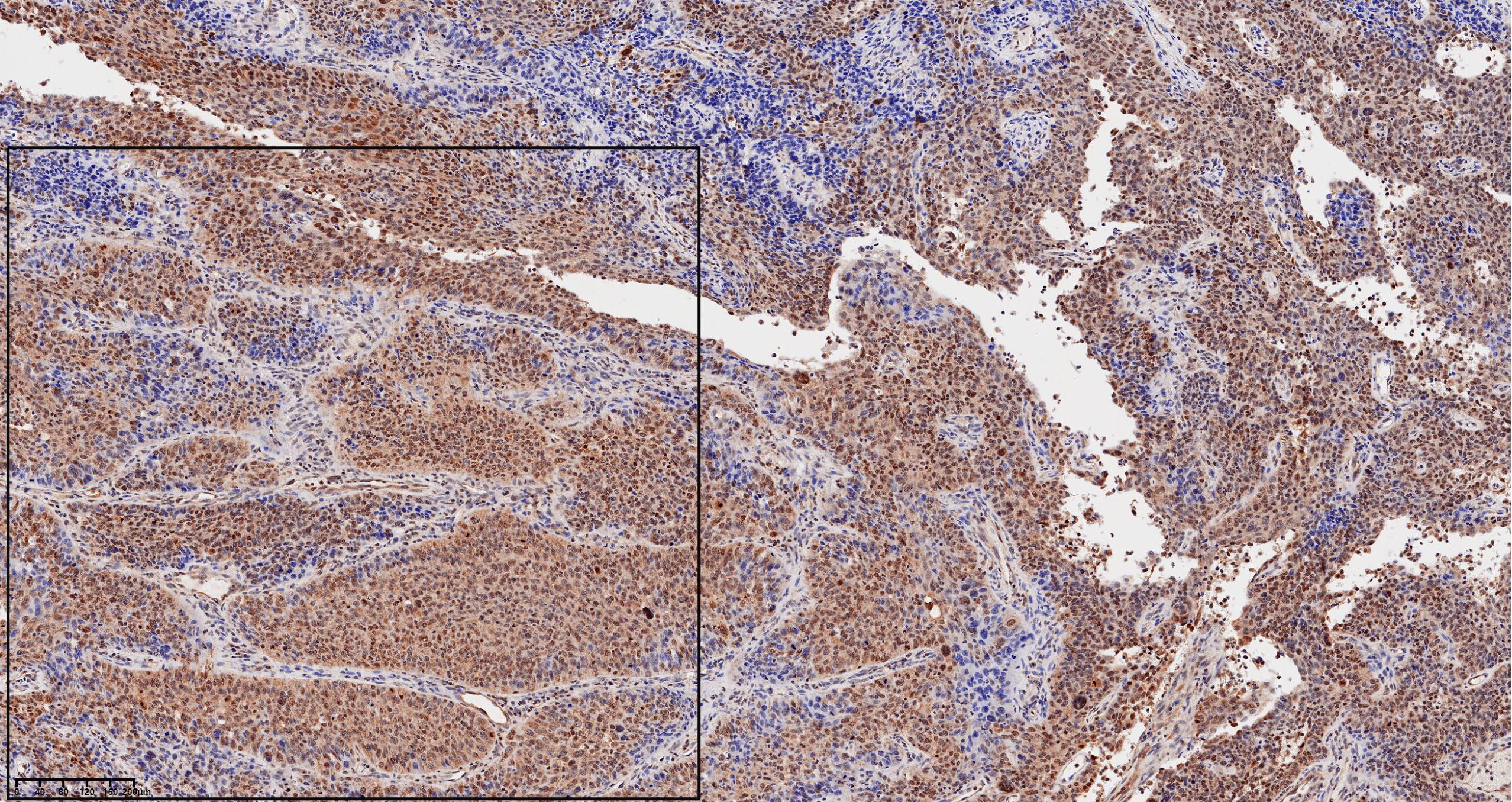

Supplement: Supplementary file 8 [file DataSheet1.zip › original data/3.qPCR+IHC+Clinical data from our hospital/2IHC (Due to ethical requirements, only partial data can be presented)/C4LYL LAMA4_9.75X.jpg]

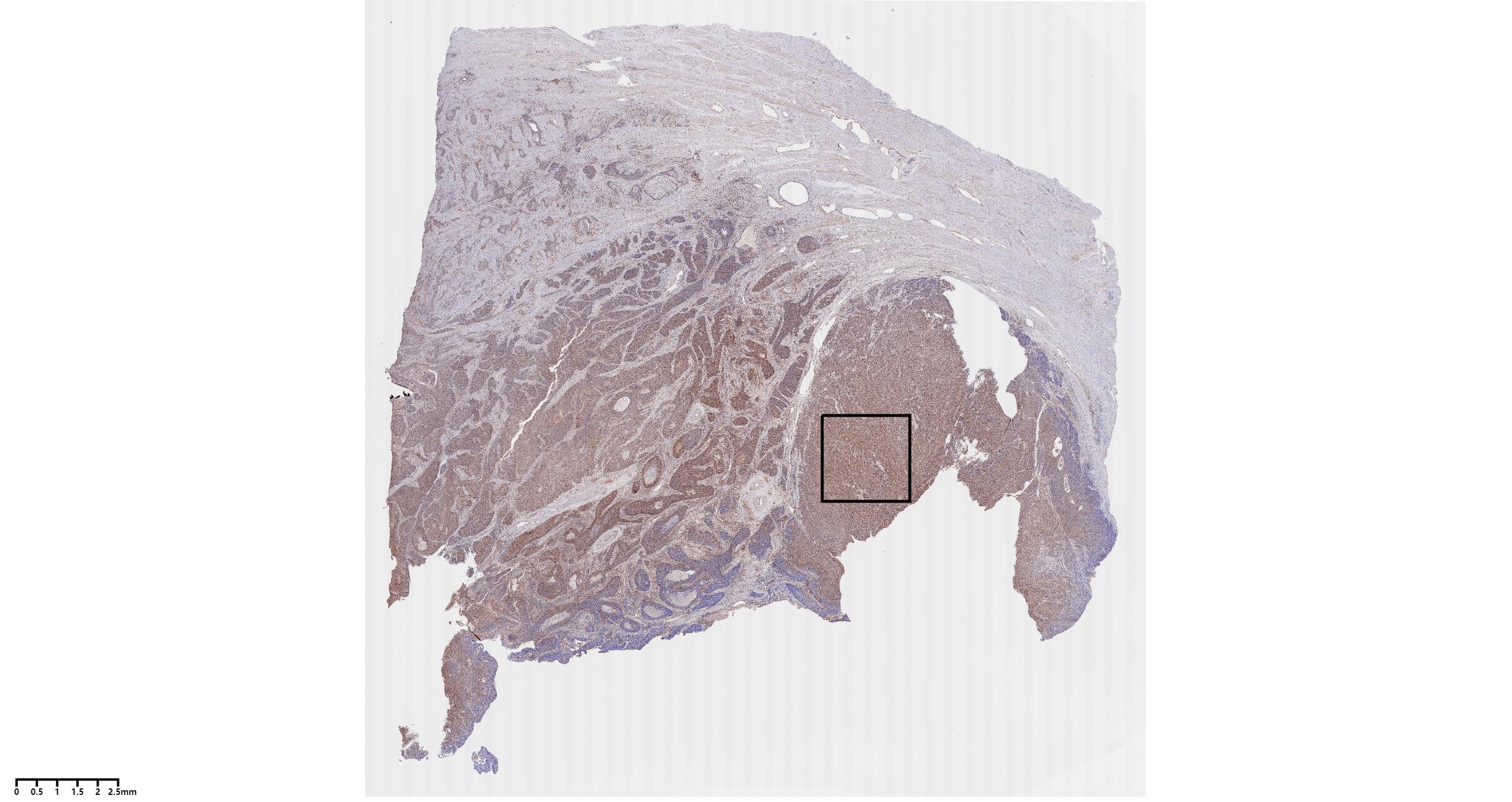

Supplement: Supplementary file 8 [file DataSheet1.zip › original data/3.qPCR+IHC+Clinical data from our hospital/2IHC (Due to ethical requirements, only partial data can be presented)/C6WJ LAMA4_0.68X.jpg]

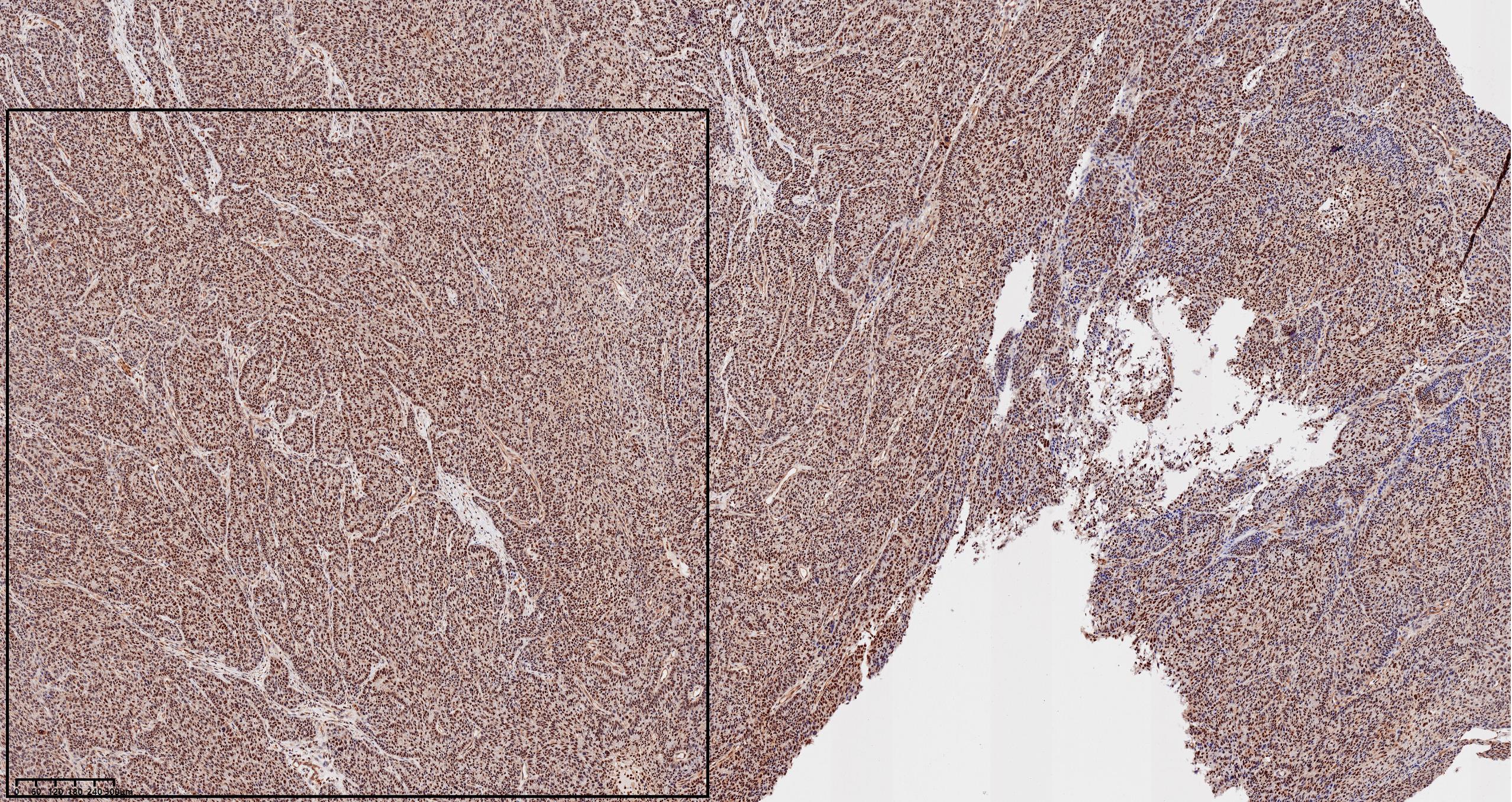

Supplement: Supplementary file 8 [file DataSheet1.zip › original data/3.qPCR+IHC+Clinical data from our hospital/2IHC (Due to ethical requirements, only partial data can be presented)/C6WJ LAMA4_5.41X.jpg]

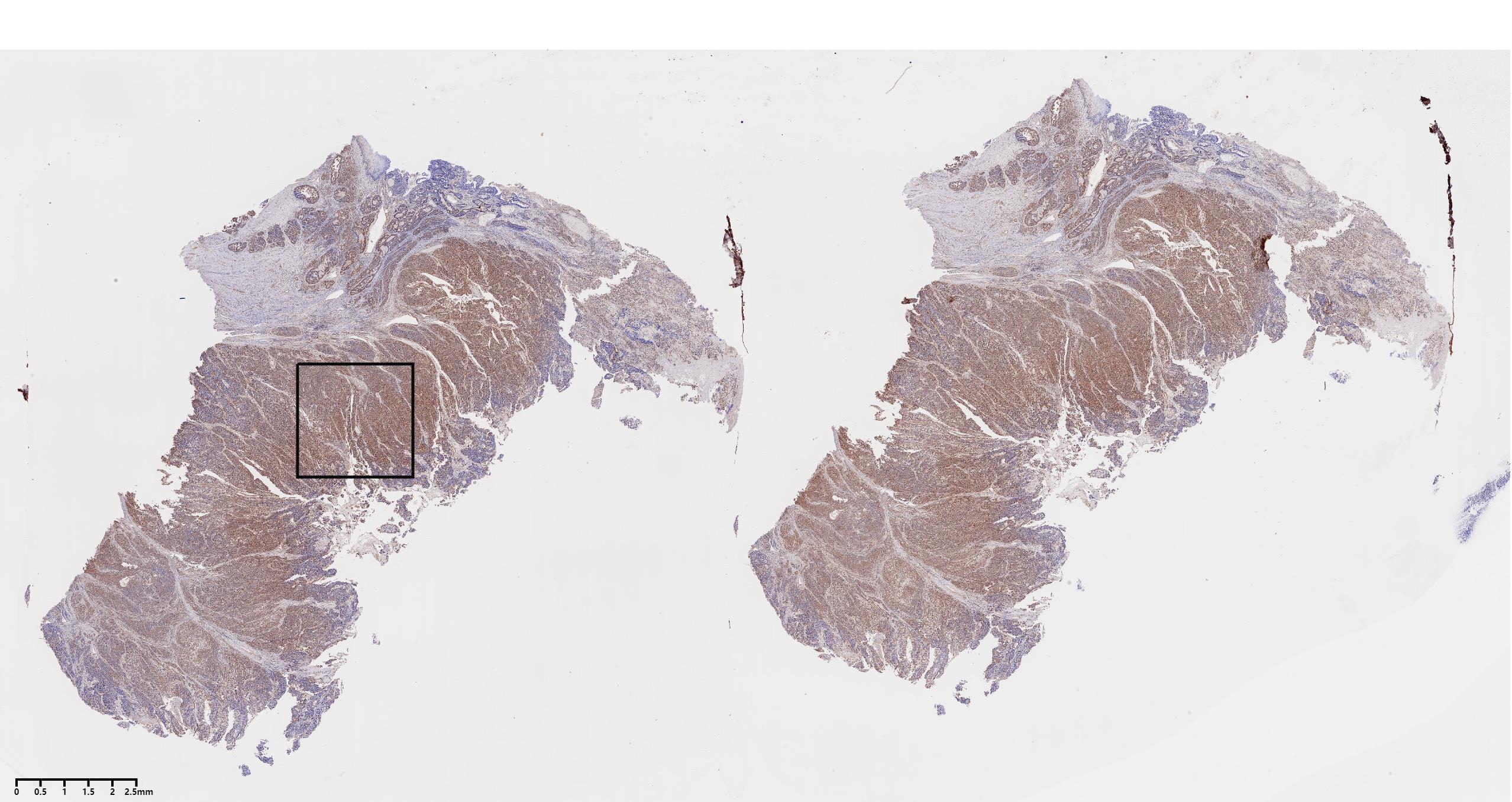

Supplement: Supplementary file 8 [file DataSheet1.zip › original data/3.qPCR+IHC+Clinical data from our hospital/2IHC (Due to ethical requirements, only partial data can be presented)/FC1LLJ LAMA4_0.80X.jpg]

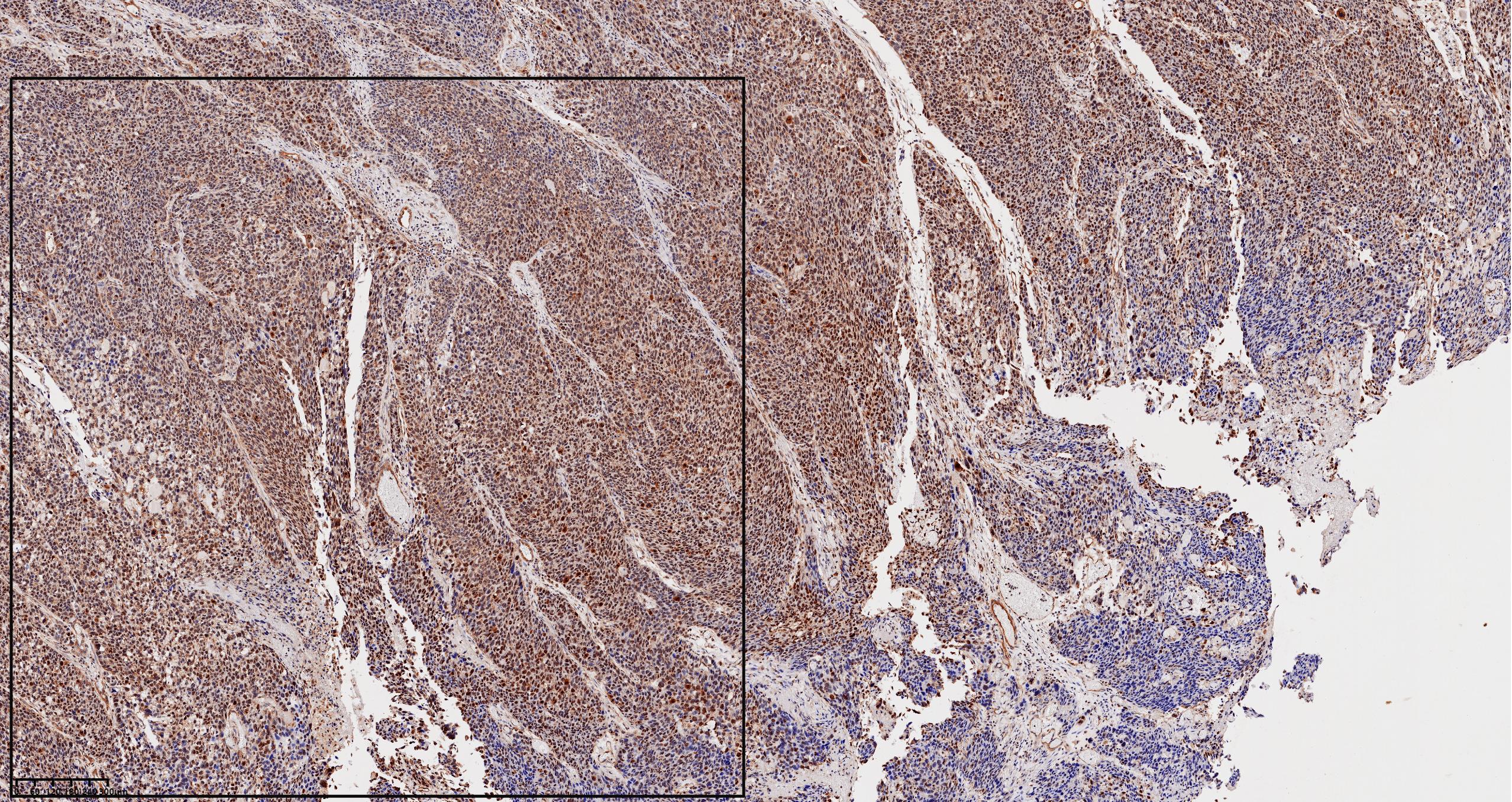

Supplement: Supplementary file 8 [file DataSheet1.zip › original data/3.qPCR+IHC+Clinical data from our hospital/2IHC (Due to ethical requirements, only partial data can be presented)/FC1LLJ LAMA4_5.06X.jpg]

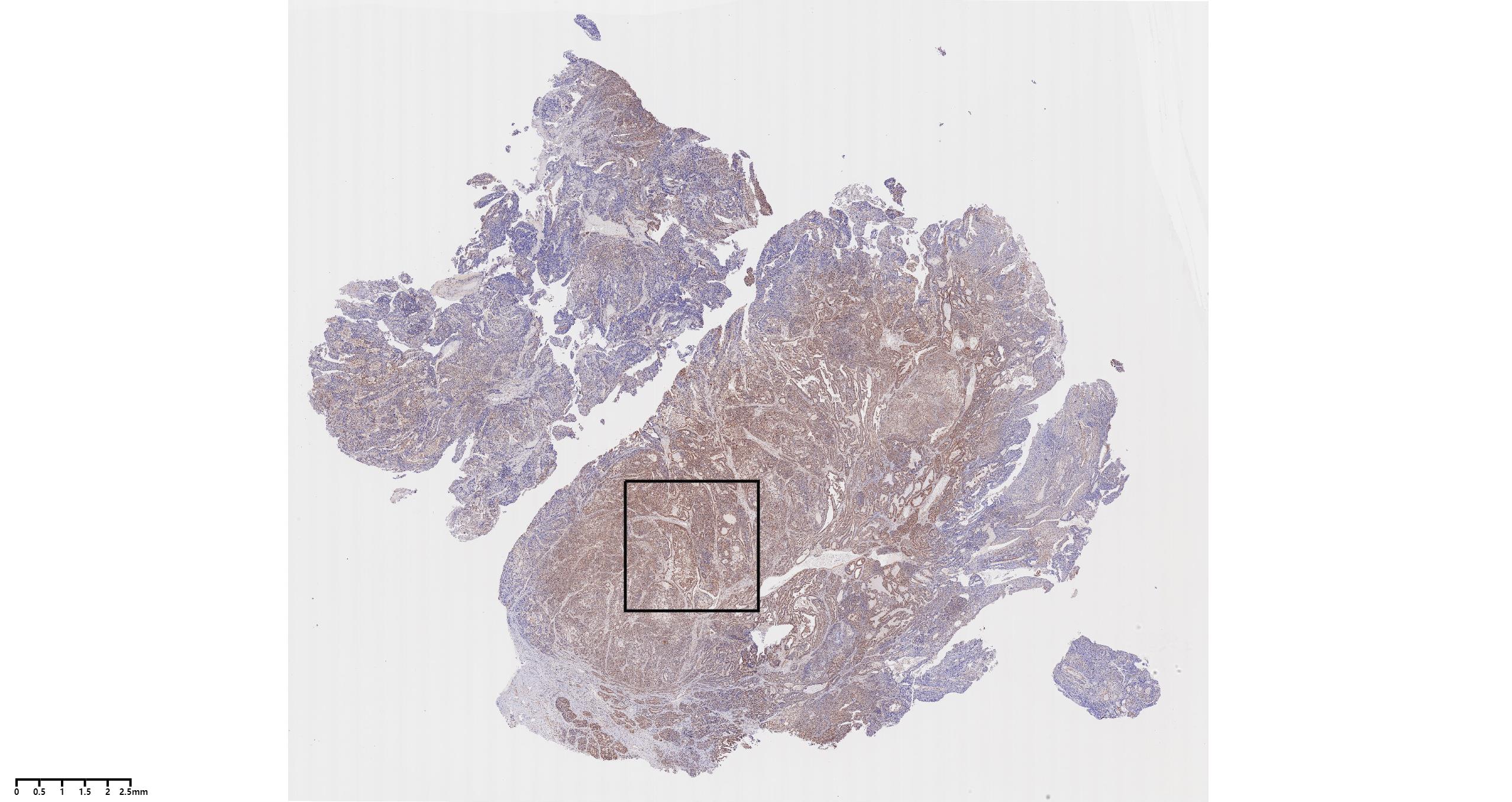

Supplement: Supplementary file 8 [file DataSheet1.zip › original data/3.qPCR+IHC+Clinical data from our hospital/2IHC (Due to ethical requirements, only partial data can be presented)/FC1LLJZ LAMA4_0.76X.jpg]

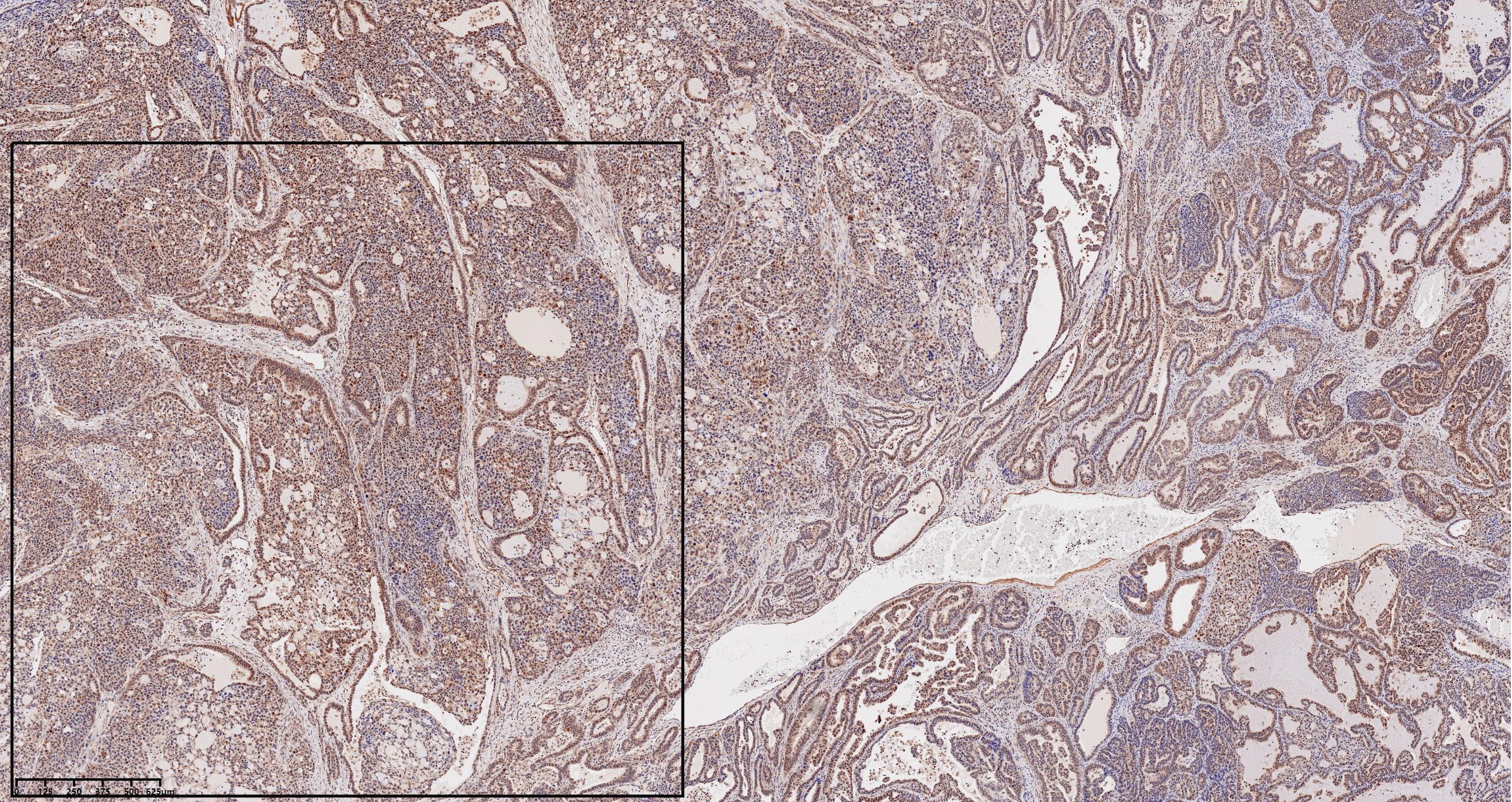

Supplement: Supplementary file 8 [file DataSheet1.zip › original data/3.qPCR+IHC+Clinical data from our hospital/2IHC (Due to ethical requirements, only partial data can be presented)/FC1LLJZ LAMA4_3.82X.jpg]

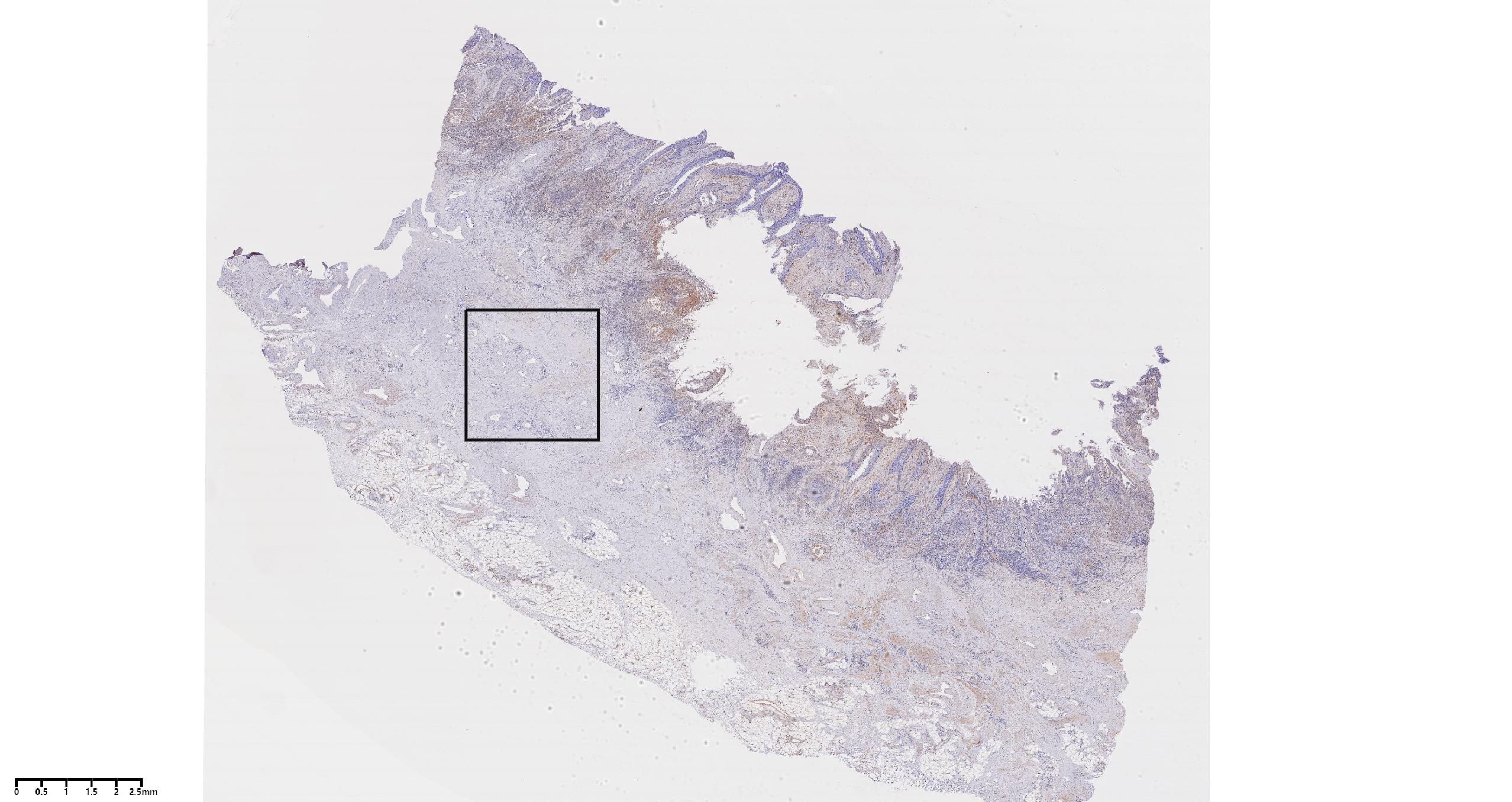

Supplement: Supplementary file 8 [file DataSheet1.zip › original data/3.qPCR+IHC+Clinical data from our hospital/2IHC (Due to ethical requirements, only partial data can be presented)/FC2WXC-15 LAMA4_0.83X.jpg]

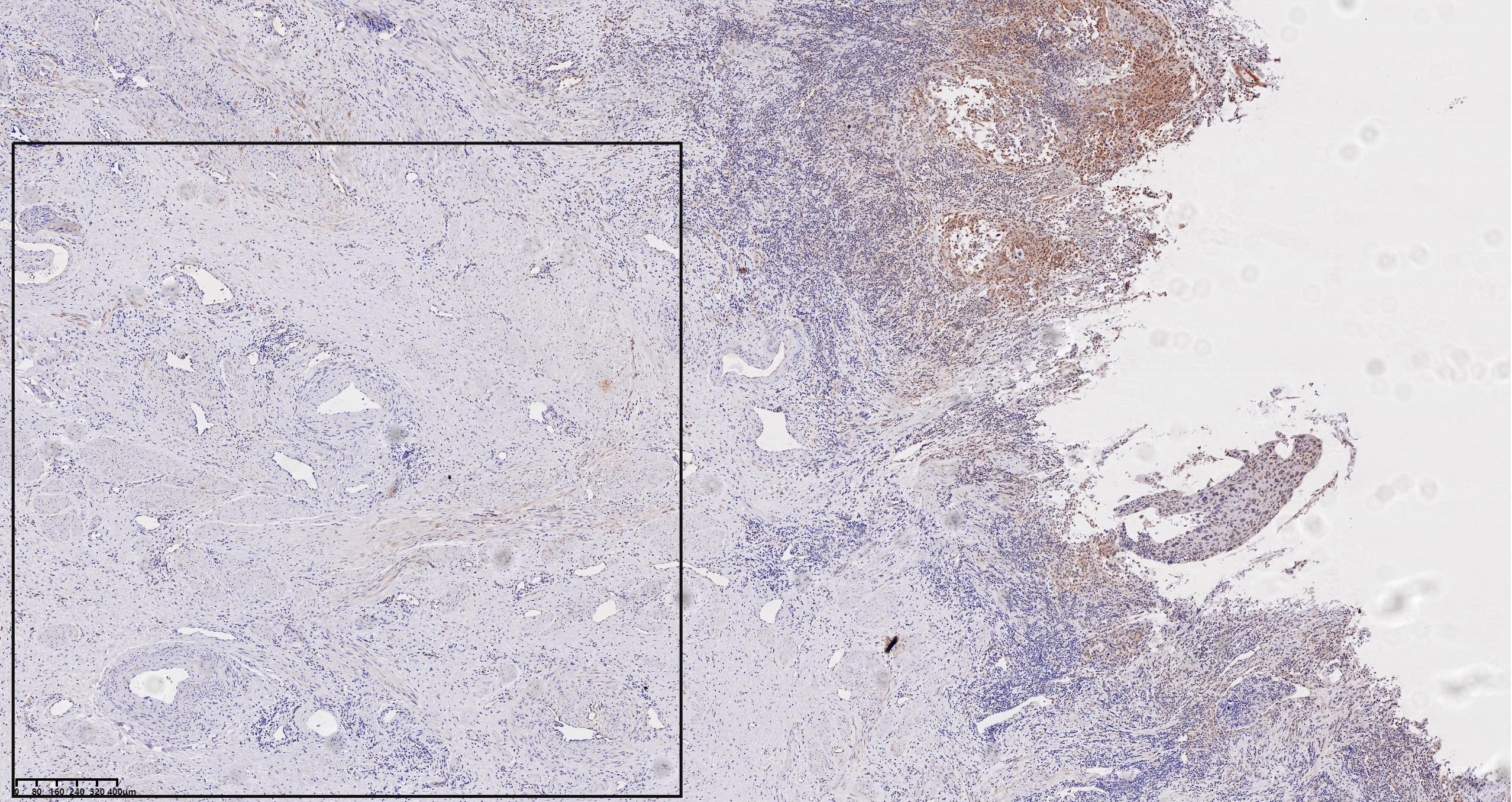

Supplement: Supplementary file 8 [file DataSheet1.zip › original data/3.qPCR+IHC+Clinical data from our hospital/2IHC (Due to ethical requirements, only partial data can be presented)/FC2WXC-15 LAMA4_4.19X.jpg]

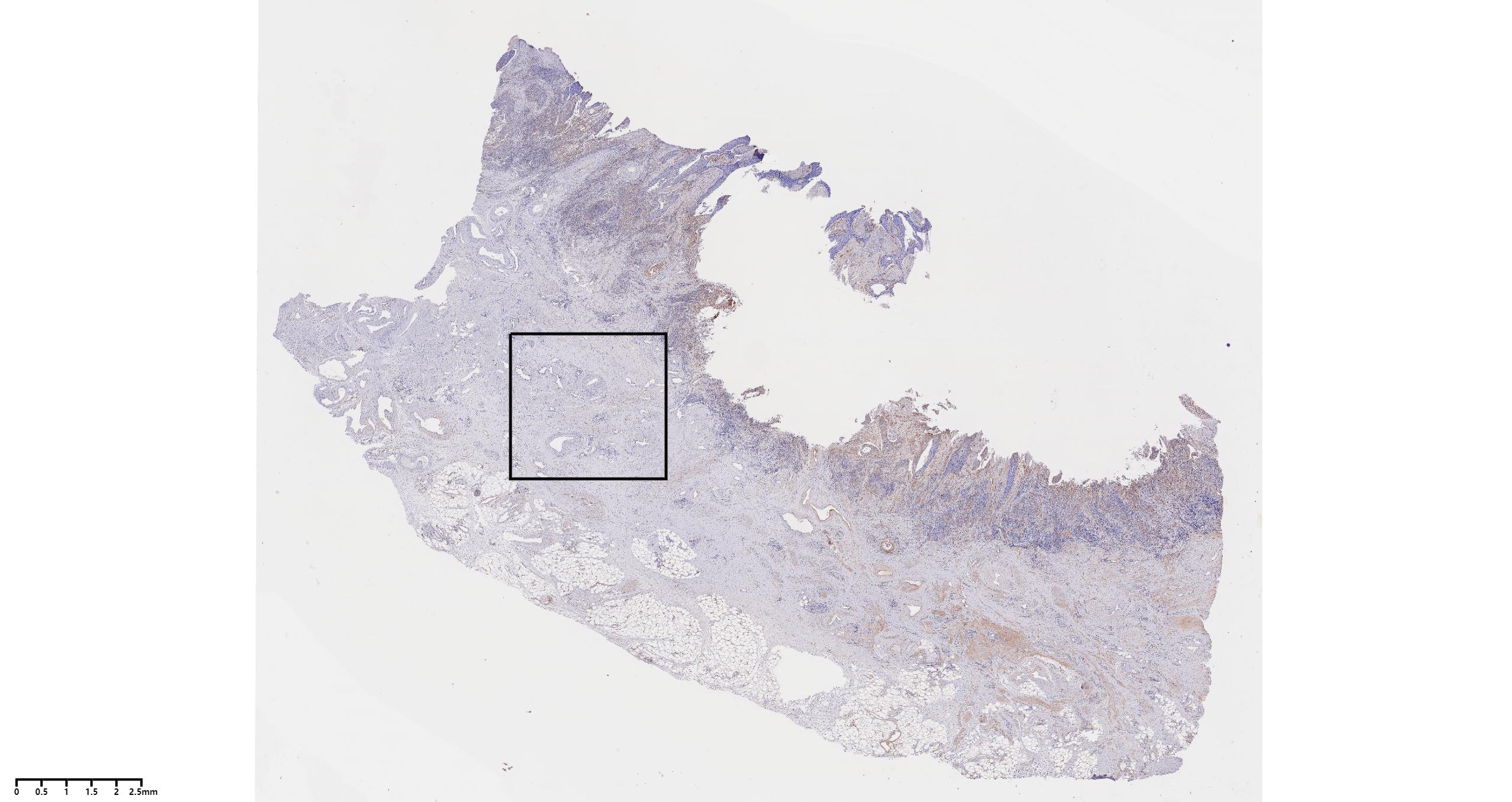

Supplement: Supplementary file 8 [file DataSheet1.zip › original data/3.qPCR+IHC+Clinical data from our hospital/2IHC (Due to ethical requirements, only partial data can be presented)/FC2WXC-2 LAMA4_0.83X.jpg]

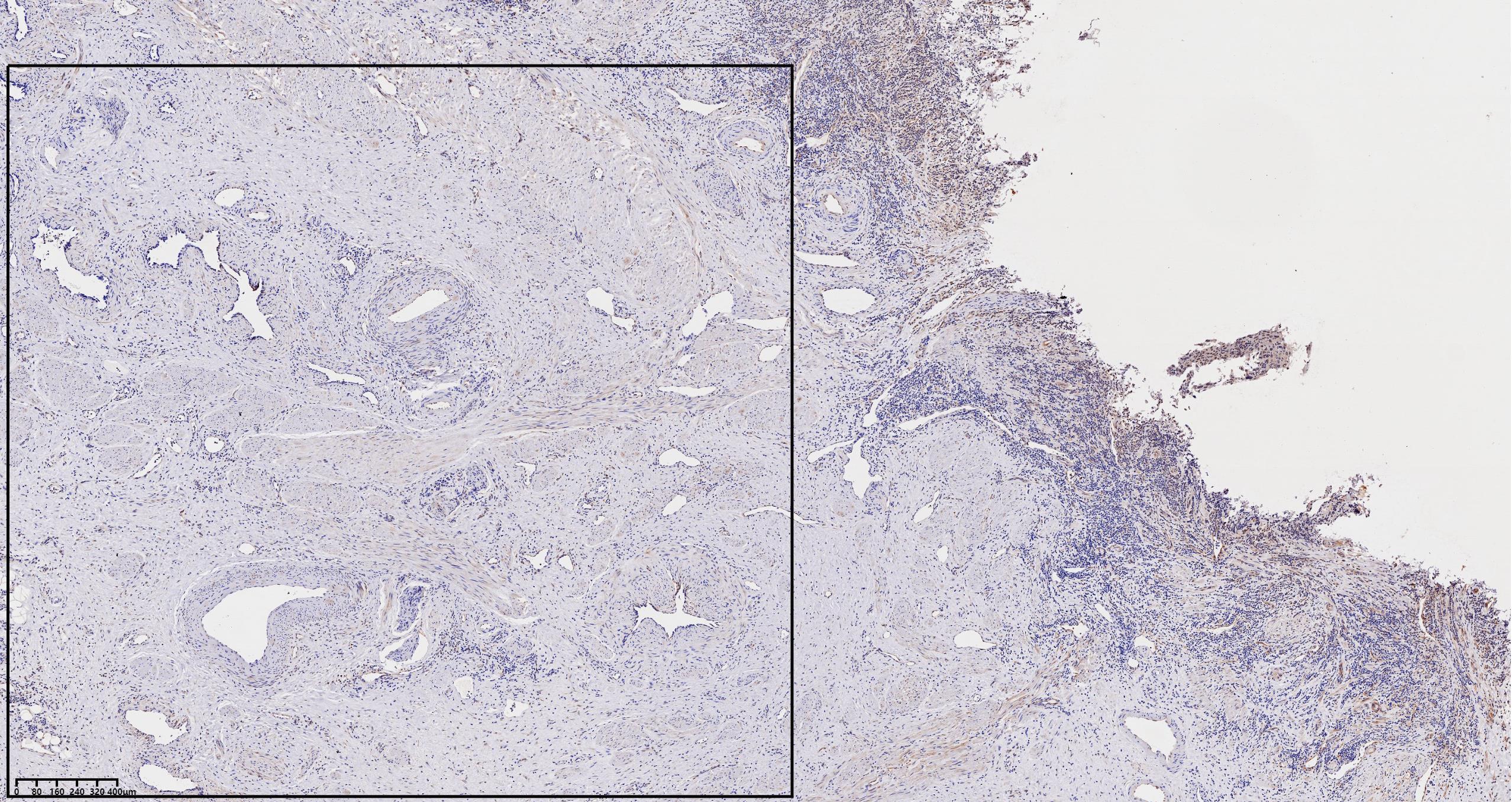

Supplement: Supplementary file 8 [file DataSheet1.zip › original data/3.qPCR+IHC+Clinical data from our hospital/2IHC (Due to ethical requirements, only partial data can be presented)/FC2WXC-2 LAMA4_4.19X.jpg]
